# Decoding Cuproptosis-Sphingolipid-Immune Crosstalk in Atopic Dermatitis: A Multi-Omics Network Analysis

**DOI:** 10.3390/biomedicines13061349

**Published:** 2025-05-31

**Authors:** Xiaowen Wen, Shulin Jia, Jing Wu, Suitian Wang, Teng Yu, Haoyou Xu

**Affiliations:** 1 Department of Dermatology, Shenzhen Hospital (Futian) of Guangzhou University of Chinese Medicine, Shenzhen 518034, China; xiaowen@gzucm.edu.cn (X.W.); wendyshulin@163.com (S.J.); 18129836624@163.com (J.W.); 18818577987@163.com (S.W.); ytfish110@126.com (T.Y.); 2 Department of Neurology, Guangdong Provincial Hospital of Chinese Medicine, The Second Affiliated Hospital of Guangzhou University of Chinese Medicine, Guangzhou 510120, China

**Keywords:** atopic dermatitis, cuproptosis, immune infiltration, bioinformatics, sphingolipid metabolism

## Abstract

**Background:** This study pioneers the exploration of the role of cuproptosis (a novel form of regulated cell death) in the pathogenesis of atopic dermatitis (AD). **Methods:** We integrated two datasets (GSE157194 and GSE193309) from the GEO database and employed weighted gene co-expression network analysis (WGCNA) to identify disease-related modules. Through multi-dimensional approaches, including differential gene expression analysis, functional enrichment analysis, GeneMANIA network construction, GSEA/GSVA pathway enrichment analysis, and immune infiltration analysis, we systematically elucidated the regulatory mechanisms of cuproptosis-related genes (CRGs) in AD. **Results:** The findings reveal novel mechanisms underlying AD pathogenesis. We identified 14 co-expression modules and 1173 differentially expressed genes, among which SPTLC2, AMD1, and IGSF3 were identified as key hub genes (AUC > 0.75). In-depth mechanistic analysis uncovered critical pathophysiological features of AD, including significant enrichment in chemokine signaling pathways (*p* < 0.001) and copper-dependent metabolic reprogramming. Notably, immune infiltration analysis demonstrated abnormal activity in 20 out of 21 immune cell types, particularly Th2 cells and macrophages, which showed strong correlations with CRG expression patterns. These findings establish an innovative “metabolic checkpoint” model for AD progression, highlighting dysregulation of the sphingolipid-immune axis as a key pathogenic mechanism. **Conclusions:** This study provides novel evidence, suggesting a potential link between AD and copper metabolism dysregulation, and identifies several promising targets that may aid in diagnosis and treatment. Our findings contribute to the growing understanding of AD pathogenesis and hint at possible new therapeutic directions, including copper chelation or sphingolipid-modulating approaches for difficult-to-treat AD cases. The identified CRG signatures may serve as potential biomarkers and therapeutic targets for personalized management strategies of this complex skin disorder.

## 1. Introduction

Atopic Dermatitis (AD) is a common, heterogeneous, complex, and inflammatory skin disease characterized by persistent itchy skin and eczema-like rashes as typical skin lesion symptoms. Its phenotype and clinical course are diverse and complex. Based solely on disease manifestations, the global lifetime prevalence is approximately 20% [1], with most cases occurring in early childhood [2]. When considering factors such as family history, subclinical cases, and comorbid allergic diseases [3,4], the prevalence is even higher. Long-term recurrent skin lesions and itching severely affect children’s growth and development [5], adult sleep [6], and mental state [7,8]. The resulting treatment needs and economic burden of the disease [9] have become serious public social issues. Despite numerous research reports on AD, there are still many unknowns about its complete pathophysiological process. Various treatment guidelines only focus on relieving symptoms and are ineffective in preventing the pathophysiology and comorbidities of AD, limiting treatment options [10]. Therefore, there is an urgent need to deeply understand various pathogenic factors and their interactions, elucidate the pathogenesis of AD, and break through the treatment dilemma of AD. Technologies such as bioinformatics and bioinformatics analysis provide new opportunities for this endeavor.

The pathogenesis of AD is complex and multifactorial, with skin barrier dysfunction serving as the initial step in the development of AD [11,12], while imbalance and the itch-scratch cycle are critical factors in disease progression and chronicity [8]. Trace elements are essential for preserving the structural integrity and normal function of the skin barrier, and both deficiencies and excesses can trigger epidermal cell death [13,14]. Almost all types of cells use copper in various physiological processes. The accumulation of copper within cells can trigger oxidative stress and disrupt cellular functions; thus, the homeostasis of copper is strictly regulated. Investigating the regulation of mitochondrial copper homeostasis under both normal and pathological conditions can serve as a new therapeutic target for inflammatory diseases [15]. Nonetheless, the connection between copper and AD remains poorly understood. Earlier clinical cohort research has discovered that children with AD exhibit notably elevated serum copper and ceruloplasmin concentrations compared to healthy peers [16], with comparable alterations also noted in copper levels in hair and umbilical cord blood [17,18]. However, recent meta-analyses have not shown a significant correlation between copper and AD [19]. This suggests that while an association between copper and AD can be observed, the relationship between the two cannot be definitively determined, and the relationship between copper-related skin barrier and immune infiltration has not been fully explored. Therefore, the current evidence regarding the possible mechanism of copper-mediated AD pathogenesis is very limited.

In 2022, the Todd R. Golub laboratory first proposed cuproptosis, a novel cell death modality that offers a fresh perspective on copper homeostasis in the progression of AD. Cuproptosis induces mitochondrial-dependent cell death through a copper overload mechanism, which begins with the accumulation of copper within mitochondria. The interaction of Cu^2+^ ions with lipoylated enzymes leads to the aggregation of lipoylated proteins and impairment of iron-sulfur cluster biogenesis. These molecular disturbances activate a cascade of proteotoxic stress responses, ultimately resulting in irreversible apoptosis [20,21]. Cuproptosis represents a distinct type of cell death, fundamentally different from other forms such as ferroptosis and apoptosis. Although the precise mechanism of cuproptosis in AD remains unclear, studies have suggested that copper metabolism and copper-related proteins may play critical roles in skin immune regulation [22,23]. A unique subset of pyroptosis-sensitive keratinocytes has been identified in the skin of individuals diagnosed with AD [24], and there may be common regulatory genes between cuproptosis and ferroptosis [25,26,27]. This suggests that cuproptosis has the potential to become a novel therapeutic target for the treatment of AD. Exploring the underlying pathological mechanisms of cuproptosis in AD through the application of bioinformatics algorithms and tools may facilitate the discovery of biomarkers and enable precision therapy. Currently, there are no reported in-depth studies on the potential pathological mechanisms of cuproptosis in the pathogenesis of AD.

This study explores the pathophysiology of AD from the perspective of cuproptosis, utilizing bioinformatics analysis. We identified gene co-expression modules or obtained hub genes through weighted gene co-expression network analysis (WGCNA) to further clarify the connections between cuproptosis-related genes (CRGs) and immune infiltration, as well as key signaling pathways. Additionally, we validated the capability of hub genes to serve as diagnostic biomarkers for AD by employing receiver operating characteristic (ROC) curves. A deeper understanding of cuproptosis in AD progression can guide future research on its pathogenesis and treatment.

## 2. Methods

**Figure 1:**
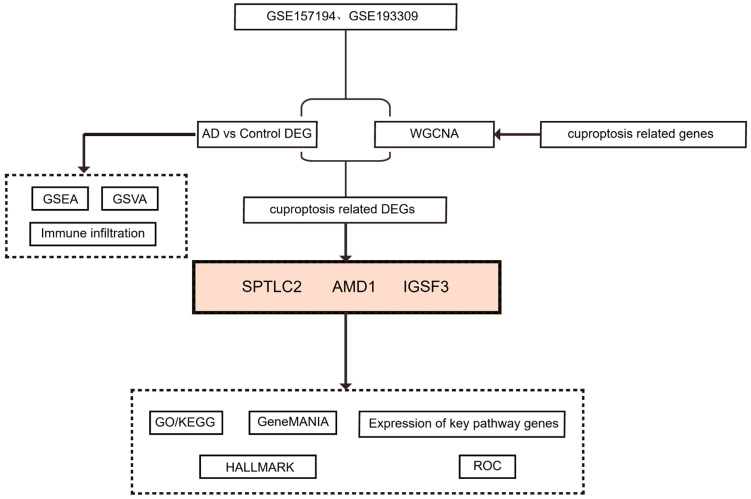
Study flowchart. Utilizing whole-genome data for AD from the GEO database (GSE157194 and GSE193309 were merged for all subsequent analyses), we conducted differential analysis and applied WGCNA to identify differentially expressed genes associated with cuproptosis (Cu-DEGs). This was followed by an integration of GO/KEGG enrichment analysis, gene interaction network construction, and validation of key pathways associated with hallmark features, ROC curve evaluation, GSEA/GSVA analysis, and immune infiltration profiling. Through this comprehensive approach, we systematically untangled the regulatory mechanisms of Cu-DEGs in AD and assessed their diagnostic potential.

The study’s workflow is shown in Figure 1.

### 2.1. Data Acquisition and Preprocessing

This article is based on data sourced from the Gene Expression Omnibus (GEO) public database https://www.ncbi.nlm.nih.gov/geo/ (accessed on 1 October 2024). The atopic dermatitis genome expression profile was obtained and downloaded from the GEO database utilizing the R software (v4.1.0) package GEO query on 1 October 2024. We used the keywords ‘atopic dermatitis’, ‘human beings’, ‘peripheral blood’, and ‘expression profiling by array’ to make sure that every group had over 10 subjects. Fifty-seven whole blood specimens were collected from AD patients in GSE157194, and in GSE193309, 97 samples were collected, with 49 of them coming from AD patients and 48 from healthy controls (HC).

In the actual analysis, we merged the GSE157194 dataset (comprising skin samples from 57 AD patients) with the GSE111154 dataset (comprising skin samples from 49 AD patients and 48 healthy controls) to generate a novel dataset containing skin samples from 106 AD patients and 48 healthy controls (i.e., the training set), which was used for all analyses in this study. The ComBat method implemented in the ‘sva’ package in R was employed to rectify batch effects arising from non-biotechnological variations. To assess the efficacy of this correction, principal component analysis (PCA) was performed (Supplementary Figure S1). The GSE232127 dataset (comprising 21 skin samples from AD patients and 9 control samples) was used as an external validation set in this study to evaluate the generalizability of the analysis results. This study adhered to the data access policies of each database and primarily utilized publicly available datasets; thus, no additional ethical review was required.

Genes related to cuproptosis were extracted from the publication deposited by Tsvetkov et al. [20].

### 2.2. Analysis of AD-Related Differences

To identify differences in gene expression (DEGs) between the control group and the AD group, we employed the R package ‘limma (version 3.50.0)’ [28]. Differentially expressed genes were identified using |log2(fold change)| > 0.5 and *p* < 0.05 as thresholds. Heat maps were constructed utilizing the R package pheatmap, while clustering was performed through the application of Euclidean distance and hierarchical clustering techniques.

### 2.3. Enrichment Analysis of GSEA and GSVA

Gene Set Enrichment Analysis (GSEA) was operated using the R package “clusterProfiler (version 4.2.2)” to investigate the concordance of variations between established gene sets and biological conditions [29], while the reference gene set employed for this analysis was c2.cp.kegg.v7.5.1. symbols, sourced from the Molecular Signatures Database (MSigDB, https://www.gsea-msigdb.org/gsea/msigdb, accessed on 10 November 2024) [29,30,31]. All genes were organized based on their log2 fold change value, with the random sample count set at 1000. Significant enrichment was determined by *p* < 0.05.

The expression profiles corresponding to datasets GSE157194 and GSE193309 were analyzed via the Gene Set Variation Analysis (GSVA) package (version 1.42.0) within the R programming environment. The gene sets referenced for this analysis were derived from the “c2.Cp.Kegg.7.5.1.Symbols” collection, available in the MSigDB database. The resultant data were illustrated using the pheatmap package (version 1.0.12) within the R programming environment. Furthermore, the ssGSEA function in the GSVA package calculates scores for 50 MSigDB gene sets across multiple samples. Subsequently, a comparative analysis of the GSVA scores for distinct gene sets was performed between the control and AD groups, utilizing the Limma package for the analysis.

### 2.4. WGCNA Analysis and Identification of Significant Modules

In the present investigation, we employed the gene expression datasets GSE157194 and GSE193309 to develop a weighted gene co-expression network that links AD with cuproptosis-related genes. This was accomplished through the application of the WGCNA (version 1.70-3) package within the R programming environment [32]. Furthermore, we examined the associations between the identified gene modules and the clinical phenotypes associated with AD.

Pearson correlation assessed gene expression similarity, and PickSoftThreshold with β = 8 enhanced co-expression to create a weighted adjacency matrix and scale-free network. Using a hierarchical clustering method, the same gene modules were identified and categorized by color. The dynamic tree-cutting method was used to determine the characteristics of different modules, wherein the adjacency matrix was converted into a topological overlap matrix (TOM), and then gene module clustering analysis was used to determine the target modules. Finally, the correlation between module feature vectors and cuproptosis was calculated using Pearson correlation analysis, which identified significant hub genes. Simultaneously, the architecture of the aforementioned co-expression modules was represented through topologically overlapping gene network heat maps. Furthermore, the interconnections among modules were illustrated via hierarchical clustering of eigenvector networks and heatmaps. Differentially expressed genes associated with cuproptosis (cuproptosis-related DEGs) were determined through the intersection of DEGs with those genes derived from the cuproptosis modules.

### 2.5. GO and KEGG Analysis

Systematic functional analyses using biological annotation systems were carried out to delineate the molecular pathophysiology of genes that are differentially expressed in cuproptosis. Gene ontology (GO) categorization was employed to decode gene functionality across three hierarchical dimensions: biological processes (BP) involved, molecular function (MF) characterization, and cellular compartmental (CC) localization. KEGG pathway enrichment analyses were used to perform parallel functional profiling using the ‘clusterProfiler’ R package (v4.2.2) [33]. The KEGG platform served as the principal analytical framework for mapping genome–pathway–disease–pharmacological interactions, enabling precise identification of biologically perturbed pathways exhibiting statistical significance (Benjamini–Hochberg adjusted *p* < 0.05) [34]. The focus of this approach was on metabolic networks that exhibit dysregulation in cuproptosis.

### 2.6. GeneMANIA

For the functional association analysis, we examined key genes by creating protein-protein interaction (PPI) networks using the GeneMANIA platform (http://genemania.org, accessed on 15 November 2024). This online tool helps identify gene clusters that are functionally related by integrating various types of interactions, including protein–protein and protein–DNA interactions, co-expression patterns, correlations within metabolic pathways, and characteristics of spatial colocalization [35].

### 2.7. The ROC Curve

To assess the diagnostic potential of the candidate biomarkers for AD, we performed ROC curve analysis using the “pROC” package in R. This analysis allowed us to quantify the discriminative performance of the selected genes by calculating the area under the ROC curve (AUC), which serves as an objective statistical measure [36].

### 2.8. Immune Cell Infiltration and Correlation Analysis

We used single-sample Gene Set Enrichment Analysis (ssGSEA) to analyze transcriptional coordination mechanisms, measuring pathway synchronization and gene expression modulation in individual samples [37]. Immune profiling data from the Tumor-Immune System Interaction Database (TISIDB, http://cis.hku.hk/TISIDB/index.php accessed on 15 November 2024) contained comprehensive annotations for 34 distinct immunophenotypes encompassing multiple CD4+/CD8+ T lymphocyte subsets (activated, central memory, effector memory) and 28 complementary immune cell populations. Immune cell abundance indices were quantified through computational deconvolution of sample-specific expression profiles, with subsequent comparative analysis of AD versus control groups revealing differential infiltration patterns, which were graphically represented using the “ggplot2” visualization toolkit (v3.3.6) in the R environment) [38].

### 2.9. Construction of RBP-mRNA Network

We utilized the StarBase platform (https://rnasysu.com/encori/rbpDisease.php accessed on 15 November 2024) to investigate non-coding RNA interactions in a systematic manner. Our analysis involved combining CLIP-seq data with RNA degradation profiles and transcriptome-wide interaction datasets to characterize regulatory associations between messenger RNAs and ribonucleoprotein complexes. Stringent selection thresholds were implemented, requiring statistical significance (*p* < 0.05), minimal cluster density (≥5 binding clusters per target), and experimental validation through ≥5 independent CLIP-seq read counts. Following this analysis, we constructed the RBP-mRNA interaction network utilizing Cytoscape software (v3.8.2).

### 2.10. Statistical Analysis

Genomic analyses were performed using R v4.1.2 with bioinformatics packages including GEOquery, limma, WGCNA, and ggplot2. Spearman tests assessed variable correlations, while Wilcoxon and Kruskal–Wallis methods compared group differences (pairwise and multi-group, respectively). Statistical significance was defined as *p* < 0.05 (two-tailed).

## 3. Results

### 3.1. Identification of Key WGCNA Module and DEGs

**Figure 2:**
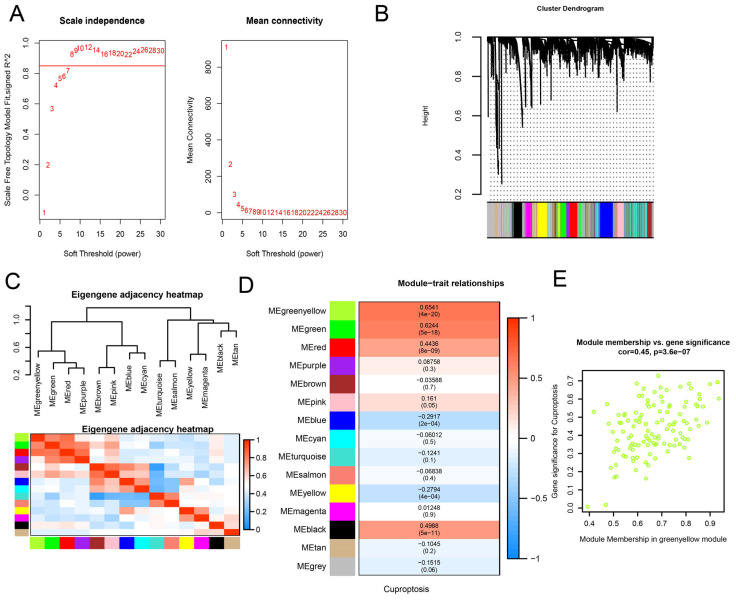
Co-expression network derived from WGCNA. (**A**) The soft threshold was set at β = 8, with the R^2^ fitting index shown. (**B**) Gene expression networks in AD revealed distinct co-expression modules. (**C**) The correlation heat map illustrates the interrelationship among modules, with rows and columns representing characteristic genes, color-coded for adjacency; red indicates high adjacency, blue low, and gradient hues illustrate intermediate interaction levels, while the diagonal red square designates the meta-module. (**D**) The association between module signature genes and cuproptosis is shown, with rows for modules and columns for features; numerical values indicate correlations, with *p*-values in parentheses, visually represented by a color-coded legend. (**E**) The correlation between module membership and gene significance for cuproptosis-related genes in the greenyellow module, where cor represents the absolute correlation coefficient between gene significance and module membership.

The WGCNA was employed to analyze genes that are associated with cuproptosis and discovered the optimal soft threshold value (β) of eight (Figure 2A). This resulted in the identification of 14 co-expression modules, without unrelated genes in the gray module (Figure 2B). To investigate inter-module relationships, we calculated the correlations between module eigengenes (MEs) and presented these correlations using a heatmap (Figure 2C). Comprehensive correlation analyses of 14 module eigengenes revealed that the greenyellow module (*n* = 117, Table S1) had the strongest association with cuproptosis induction (Pearson’s r = 0.6541, *p* < 0.05), highlighting its role in copper-dependent cell death regulation for further study (Figure 2D). Therefore, our future research will mainly focus on the green-yellow module, as it could give a clearer indication of cuproptosis. Figure 2E displays the scatter plot of gene significance for cuproptosis-related genes and module membership in the greenyellow module, showing a highly significant positive correlation (cor = 0.45, *p* < 0.05). This indicates that the most central (important) elements within the greenyellow module also tend to be highly correlated with the cuproptosis-related gene signature.

### 3.2. AD-Related Differentially Expressed Genes

**Figure 3:**
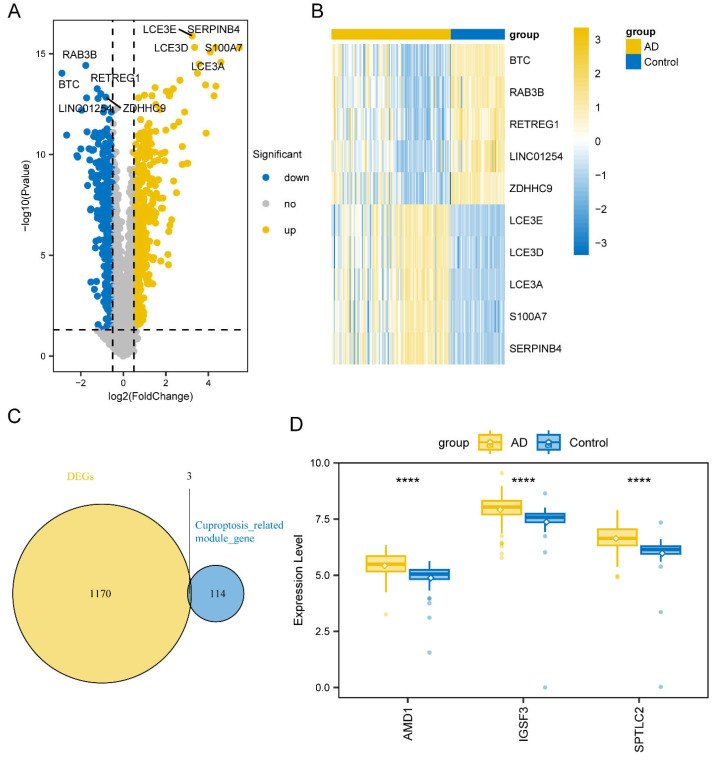
Expression of AD-related differential genes. (**A**) Volcano map shows DEGs between the AD and control samples. (**B**) Heat maps detail top DEGs. (**C**) The Venn diagram illustrates the acquisition of the hub gene. (**D**) Boxplot showing the difference in the expression of hub gene between AD and control groups (**** *p* < 0.0001).

In the transcriptomic analysis comparing AD samples to controls, researchers discovered a total of 1173 differentially expressed transcripts, including 665 up-regulated and 508 down-regulated mRNAs (Supplementary Table S2). All DEGs are shown in volcanic maps (Figure 3A), and a heatmap displays top genes (LCE3E, LCE3D, LCE3A, S100A7, SERPINB4BTC, RAB3B, RETREG1, LINC01254, ZDHHC9) (Figure 3B). Subsequently, we intersected these DEGs with the 117 genes from the greenyellow module, ultimately obtaining three genes (SPTLC2, AMD1, and IGSF3) (Figure 3C). These three genes were identified as hub genes for further investigation and exhibit significant differences in expression between groups in boxplots *p* < 0.05, Figure 3D.

### 3.3. GSEA Analysis

**Figure 4:**
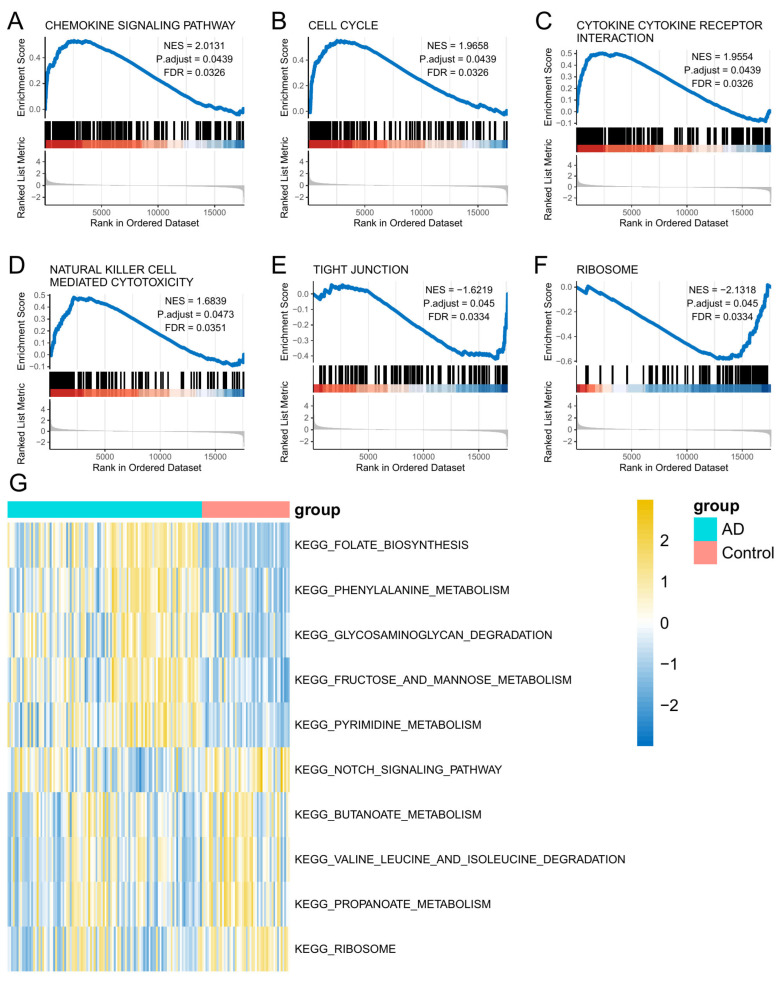
Pathways significantly enriched for AD genes. Red bars show atopic dermatitis samples, while blue bars indicate healthy controls, reflecting differential gene enrichment in pathways. (**A**) Chemokine signaling pathway. (**B**) Cell cycle. (**C**) Cytokine–receptor interaction. (**D**) NK cell-mediated cytotoxicity. (**E**) Tight junction. (**F**) Ribosome. (**G**) Visualize GSVA with heat maps.

Functional annotation analysis of differential gene expression was conducted using GSEA to identify biological processes, ranking pathways from MSigDB by normalized enrichment scores (NES) with strict criteria (adjusted *p* < 0.05, FDR < 0.05). Remarkably, seven regulatory networks showed coordinated activation: chemotactic signaling (NES = 2.013), mitotic progression (NES = 1.966), intercellular communication (NES = 1.955), immunological cytotoxicity (NES = 1.684), barrier integrity (NES = −1.622), and translational machinery (NES = −2.132), as shown in Figure 4A–F. A detailed overview of evaluation metrics and visualization matrices is in Supplementary Table S3.

### 3.4. GSVA Analysis

To characterize pathway-level alterations in AD, GSVA was performed to assess intergroup differences in biological process activation patterns. This computational approach revealed distinct metabolic pathway dysregulations, visualized through hierarchical clustering heatmaps. Two metabolic pathways, specifically phenylalanine metabolism (KEGG: hsa00360) and folate biosynthesis (KEGG: hsa00790), showed significant suppression in AD specimens compared to neurologically intact controls. In contrast, the enzymatic processes related to propanoate metabolism (KEGG: hsa00640) and branched-chain amino acid degradation pathways (KEGG: hsa00280) were notably elevated in AD cohorts. These pathway activation differences are clearly depicted in Figure 4G. By systematically quantifying variations in pathway activity using the GSVA methodology, we gain valuable insights into the metabolic reprogramming that occurs in the context of AD pathogenesis.

### 3.5. GO and KEGG Analysis

**Figure 5:**
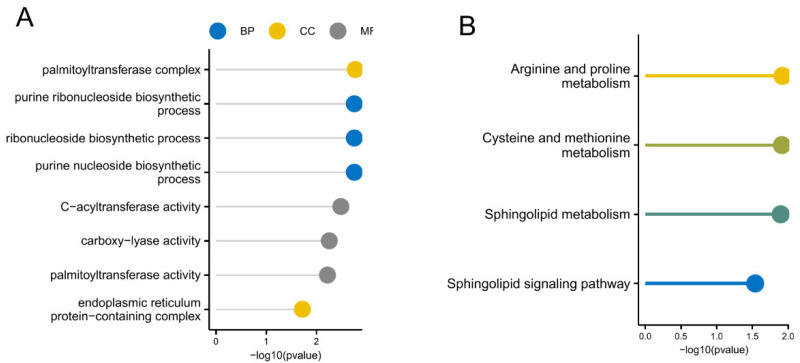
Functional enrichment of cuproptosis-linked differentially expressed genes. (**A**) GO enrichment entries, shown with a lollipop chart. The colors represent different categories: blue for Biological Process (BP), yellow for Cellular Component (CC), and gray for Molecular Function (MF). (**B**) The KEGG enrichment pathway, shown with a lollipop diagram.

Functional annotation analysis identified significant gene associations with multiple biological domains, encompassing nucleobase-containing compound biosynthesis (GO:0042451, GO:0042455, GO:0046129) within biological processes. Cellular compartmentalization patterns revealed enrichment in acyltransferase complexes (GO:0002178) and endoplasmic reticulum macromolecular assemblies (GO:0140534). Molecular function characterization demonstrated catalytic specializations in C-acyl transfer (GO:0016408), carboxylase-mediated reactions (GO:0016831), and lipid-modifying enzymatic activities (GO:0016409), as systematically visualized in Figure 5A. Pathway enrichment examinations revealed critical metabolic networks regulating nitrogenous compound homeostasis, notably arginine-proline interconversion (hsa00330) and sulfur-amino acid processing (hsa00270), along with sphingolipid catabolic/anabolic equilibrium (hsa00600), with comprehensive pathway-visualization matrices presented in Figure 5B.

### 3.6. Hub Gene Interaction Analysis

**Figure 6:**
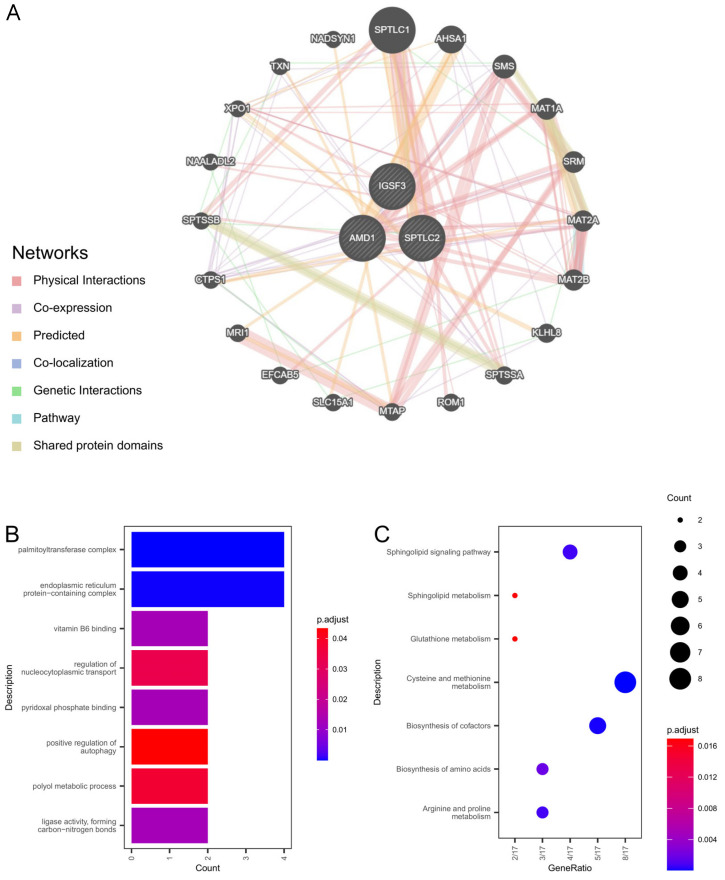
Hub gene interaction networks. (**A**) Co-expression. (**B**) Functional. (**C**) Pathway Characterization.

Integrated network analysis of central molecular regulators was identified via three interconnected regulatory hubs through GeneMANIA-derived PPI mapping (Figure 6A), with subsequent multi-omics profiling of 23 coordinated targets revealing functional specialization in nucleocytoplasmic transport regulation (GO:0046822), polyol catabolic regulation (GO:0019751), autophagy activation (GO:0010508), palmitoylation complexes (GO:0002178), and pyridoxal phosphate/vitamin B6-mediated catalysis (GO:0030170, GO:0070279), as detailed in Figure 6B and Supplementary Table S4. Pathway enrichment demonstrated critical involvement in sulfur-amino acid homeostasis (hsa00270), sphingolipid signaling (hsa04071), and redox metabolism (hsa00480), comprehensively visualized in Figure 6C and Supplementary Table S5.

According to KEGG’s analysis, the main enrichment pathways are Cysteine and methionine metabolism (hsa00270), Biosynthesis of cofactors (hsa01240), Sphingolipid signaling pathway (hsa04071), Arginine and proline metabolism (hsa00330), Biosynthesis of amino acids (hsa01230), Sphingolipid metabolism (hsa00600), and Glutathione metabolism (hsa00480) (Figure 6C, Supplementary Table S6).

### 3.7. Validation of Key Pathway Gene Sets

**Figure 7:**
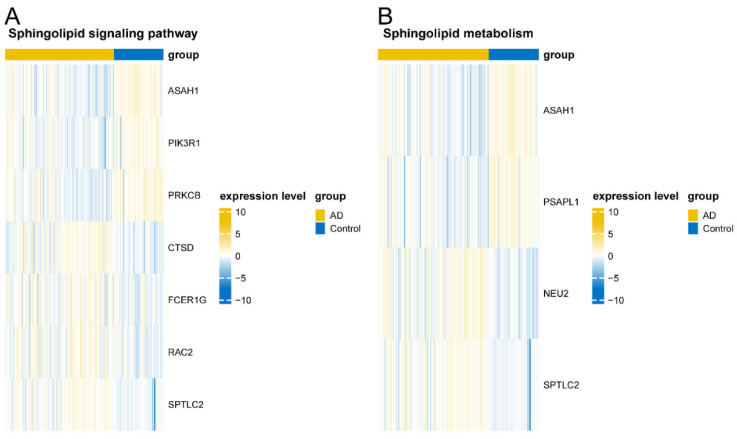
Sphingolipid signaling pathway in AD and control samples. (**A**) Sphingolipid metabolism. (**B**) Key gene expression heatmap.

Through exploring the key Sphingolipid signaling pathway and Sphingolipid metabolism of gene expression, we found that the ASAH1, PIK3R1, PRKCB, and PSAPL1 lower the expression in AD, while CTSD, FCER1G, RAC2, SPTLC2, and NEU2 showed elevated expression (Figure 7A,B).

### 3.8. Immune Infiltration Analysis

**Figure 8:**
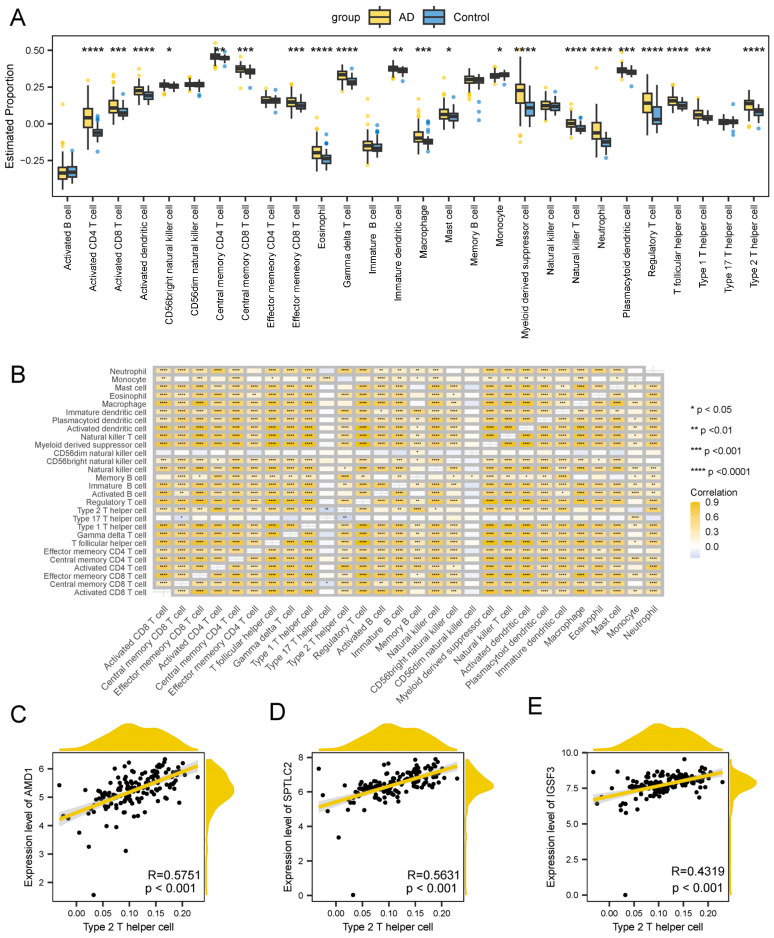
Difference in immune infiltration in AD. (**A**) Leukocyte infiltration quantification. (**B**) Correlation between immune cells. The scatter plots illustrate the correlations between AMD1 (**C**), SPTLC2 (**D**), GSF3 (**E**), and Type 2 T helper cells. The solid line represents the linear regression fit, while the shaded area corresponds to the 95% confidence interval. The asterisks indicate the *p*-values: **** *p* < 0.0001, *** *p* < 0.001, ** *p* < 0.01, * *p* < 0.05).

Given the potential pathogenic role of immune dysregulation in AD, we conducted systematic immune profiling to compare leukocyte infiltration patterns between AD and control cohorts. Quantitative deconvolution of 28 immune subtypes revealed significant intergroup disparities in 21 cell populations (*p* < 0.05), with AD specimens demonstrating marked elevation in 20 subtypes-including multiple CD8^+^ T lymphocyte subsets (activated, central memory, effector memory), Th1/Th2/Th17/Tfh polarized CD4^+^ cells, and innate immune effectors (macrophages, neutrophils, dendritic cell subsets) (Figure 8A). Network analysis identified coordinated immune cell interactions dominated by synergistic relationships (Figure 8B).

Three hub genes—AMD1, SPTLC2, and IGSF3—exhibited significant Th2 cell associations (AMD1: r = 0.575; SPTLC2: r = 0.563; IGSF3: r = 0.432; all *p* < 0.001), suggesting Th2 polarization as a potential immunoregulatory axis in AD pathogenesis (Figure 8C–E). These findings collectively implicate CD8^+^ T cell hyperactivation and Th2-skewed adaptive immunity as hallmarks of AD-associated neuroinflammation.

### 3.9. Hub Gene-Related Signaling Pathways

**Figure 9:**
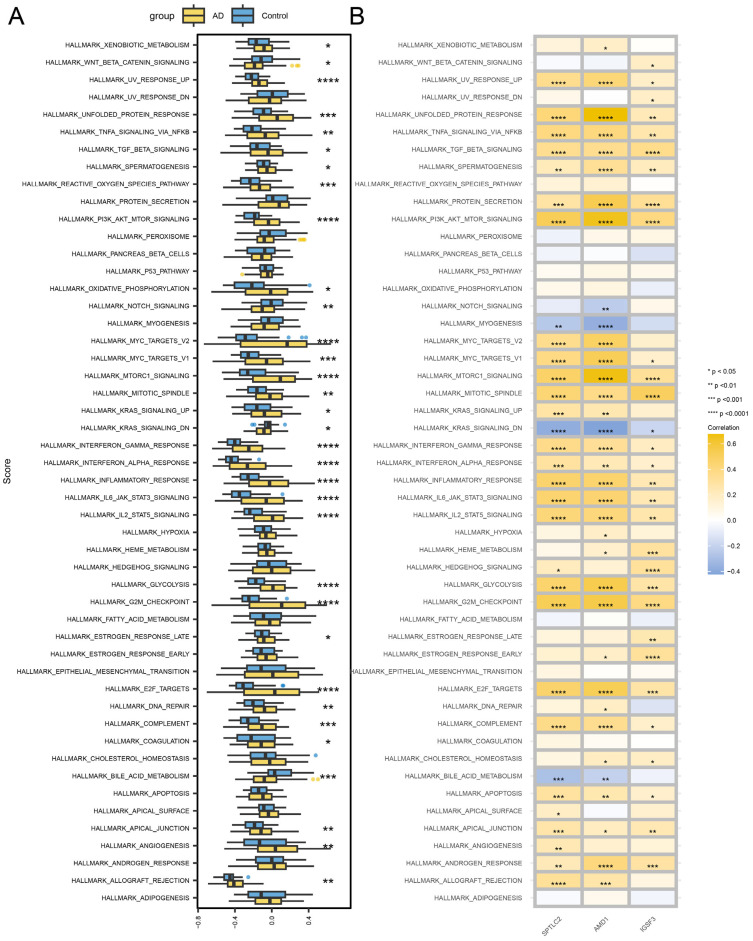
Hub gene–hallmark pathway interactions. (**A**) AD and control group comparison. (**B**) Hub gene correlation. **** *p* < 0.0001, *** *p* < 0.001, ** *p* < 0.01, * *p* < 0.05.

To investigate the molecular mechanisms associated with AD pathogenesis, GSVA was utilized to assess activity variations across 50 hallmark pathways in AD patients versus control cohorts. Comparative analysis revealed substantial activation of 29 biological pathways in AD cases, including immune-related processes (Allograft Rejection, Inflammatory Response, Interferon α/γ Responses), cellular proliferation mechanisms (G2M Checkpoint, Mitotic Spindle, E2F Targets), metabolic dysregulation (Glycolysis, Oxidative Phosphorylation, mTORC1 Signaling), and stress response pathways (Unfolded Protein Response, UV Response Upregulation). Four pathways exhibited significant suppression: Bile Acid Metabolism, Notch Signaling, Wnt/β-catenin Signaling, and KRAS Signaling Downregulation, as visually represented in Figure 9A.

Furthermore, correlation analyses were conducted to elucidate potential interactions between three key hub genes showing prominent differential expression and these dysregulated pathways, with detailed associations depicted in Figure 9B. This comprehensive approach identified critical pathway alterations involving cytokine signaling (IL6-JAK-STAT3, TNFA-NFκB), redox homeostasis (Reactive Oxygen Species), and oncogenic regulation (MYC Targets V1/V2, PI3K-AKT-mTOR), collectively suggesting multifaceted pathophysiological disruptions in AD progression.

### 3.10. Diagnostic Value of Hub Gene

**Figure 10:**
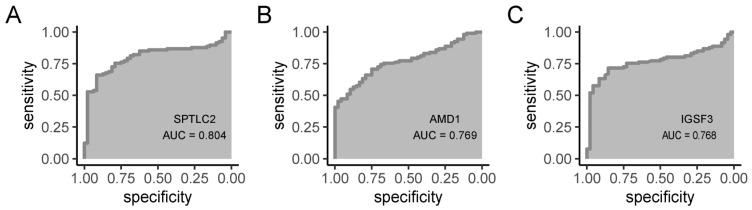
ROC curve for hub gene. (**A**) SPTLC2. (**B**) AMD1. (**C**) IGSF3.

Three central hub genes were used to perform ROC curve analysis to assess the diagnostic efficacy of potential AD biomarkers. The evaluation revealed a high degree of discriminatory power, with AUC values of 0.804 for SPTLC2, 0.769 for AMD1, and 0.768 for IGSF3 (Figure 10A–C), all surpassing the 0.75 threshold that indicates clinically relevant diagnostic accuracy. Robust AUC metrics validate both individual diagnostic efficacy and synergistic performance of a composite biomarker panel in distinguishing AD patients from controls, meeting preliminary neurological validation standards and establishing this multi-gene signature as a clinically promising detection tool.

**Figure 11:**
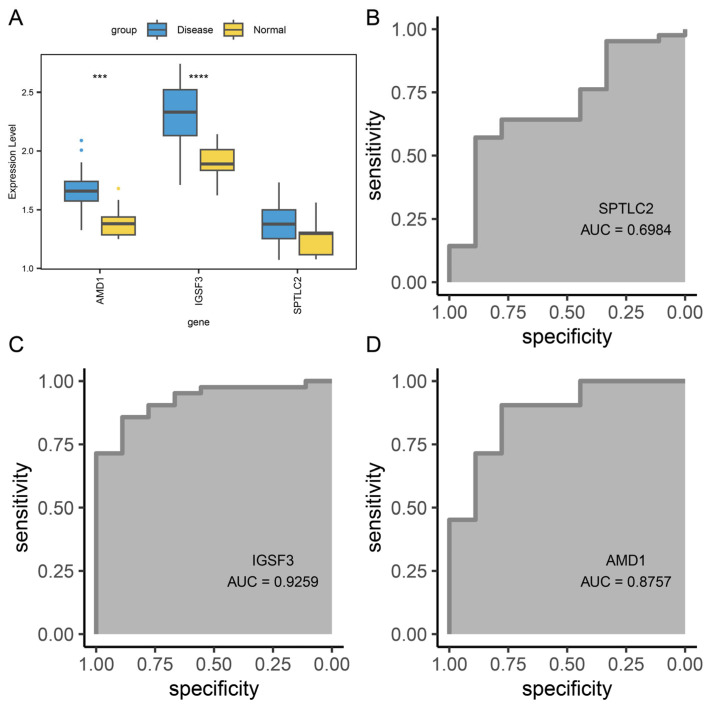
Validation of hub genes in the external independent dataset GSE232127. (**A**) Expression boxplots of the three hub genes between the disease and control groups in GSE232127. ROC curve of the hub gene SPTLC2 (**B**), AMD1 (**C**), and IGSF3 (**D**) in the external dataset GSE232127. **** *p* < 0.0001, *** *p* < 0.001.

In addition, to verify the robustness of the hub genes, we examined the expression levels of the three hub genes in the external dataset GSE232127 and evaluated their diagnostic potential for AD using receiver operating characteristic (ROC) curves. The results showed that AMD1 and IGSF3 exhibited significantly higher expression in AD and possessed excellent diagnostic potential (AUC > 0.85) (Figure 11A–D). In contrast, although SPTLC2 was also highly expressed in AD, it did not reach a statistically significant level (*p* > 0.05) (Figure 11A), which may be influenced by sample specificity and requires further validation in subsequent studies. These findings confirm the potential and application value of these hub genes as novel biomarkers for AD.

## 4. Discussion

Atopic dermatitis (AD) is an inflammatory skin disease with diverse clinical manifestations, and the global prevalence of AD is increasing each year. This increase leads to a decline in patients’ quality of life and creates a significant socio-economic burden. Currently, the main treatment options for AD include topical therapies, systemic medications, and phototherapy. However, these treatments have limitations in alleviating symptoms and enhancing patients’ quality of life, with their success varying widely among individuals. Thus, it is of great importance to further investigate the pathogenesis of AD and its related influencing factors.

This study seeks to investigate the influence of gene expression changes on cuproptosis in AD. Research has shown that cuproptosis significantly contributes to the pathogenesis of various diseases, especially in inflammation and immune responses. By employing multiple bioinformatics analysis methods, we identified 1173 DEGs and revealed the genes related to copper-induced cell death and their potential biological mechanisms in AD patients through GSEA and WGCNA. The findings offer new insights for the early diagnosis and personalized treatment of AD and set the stage for future research in this field.

Through the combined application of WGCNA and differential analysis, this study identified SPTLC2, AMD1, and IGSF3 as key genes linked to cuproptosis. There is a significant upregulation of these genes in AD patients, which will help us understand the molecular mechanisms behind AD from a genetic perspective. Cuproptosis-related genes, including SPTLC2, AMD1, and IGSF3, may collectively contribute to the progression of AD by causing metabolic changes and disrupting copper balance. SPTLC2 dysfunction in sphingolipid metabolism may compromise skin barrier integrity by disrupting ceramide-dependent stratum corneum organization [39]. Additionally, Sphingolipid metabolites can regulate copper transporters (such as ATP7A/ATP7B), and copper overload in mitochondria further inhibits the activity of sphingolipid synthase, creating a vicious cycle that amplifies oxidative stress and inflammatory responses [40]. AMD1 plays a role in this process by regulating polyamine and purine metabolism. Specifically, its involvement in spermidine synthesis consumes the glutathione precursor S-adenosylmethionine (SAM), which weakens the antioxidant defense system and makes keratinocytes more susceptible to copper-induced mitochondrial toxicity. Meanwhile, the depletion of purine nucleotides (ATP/GTP) may activate the AMPK pathway, promoting cellular energy crisis and cuproptosis [41]. Meanwhile, IGSF3’s mechanistic underpinnings remain incompletely characterized, as a member of the immunoglobulin superfamily.

As a member of the immunoglobulin superfamily, IGSF3 has not yet been fully characterized in its mechanistic underpinnings. Its abnormal expression may indirectly affect epidermal barrier integrity and copper ion transmembrane transport by disrupting cell-cell adhesion or regulating the integrin-FAK signaling pathway, thereby exacerbating inflammatory infiltration and copper-dependent cell death [42]. The combined effects of these genes suggest that the pathological features of AD, such as Th2-type inflammation, barrier defects, and chronic itching, may partly result from the interaction between sphingolipid-polyamine metabolic disorders and cuproptosis.

Analysis of pathological mechanisms revealed that central genetic regulators critically contribute to molecular pathways driving AD initiation and advancement. These pathways encompass a diverse array, including lipid metabolism and chemokine signaling. Abnormalities in the synthesis pathway of lipid metabolism-related enzymes are not only a pathological feature of AD but are also closely linked to core pathogenesis, such as skin barrier dysfunction and immune dysregulation [43]. Ceramide, along with cholesterol and free fatty acids, is a primary component of the epidermis stratum corneum permeability barrier. Studies have pointed out that changes in the ceramide molecular spectrum are directly related to the impairment of AD skin barrier permeability function [44,45]. The involvement of cuproptosis in the regulation of wound healing-related pathways has been confirmed in animal models [46]. Also enriched in the hub gene-related pathways is the chemokine signaling pathway, which regulates migration and activation of immune cells as well as inflammation. Growing evidence indicates that copper-induced cell death may play a significant pathophysiological role in inflammatory diseases [47] and immune dysregulation [40]. Based on these findings, we speculate that these hub genes may precisely regulate the synthesis of lipid metabolism-related enzymes and inflammatory responses through cuproptosis, thereby affecting the progression of AD at a deeper level.

In the analysis at the molecular level, we further uncovered the potential key roles of Sphingolipid signaling pathways and Sphingolipid metabolism in the pathogenesis of AD. As discussed earlier, we discussed how Sphingolipids regulate epidermal cell signaling and AD onset. Although current research directly involving SPTLC2, AMD1, and IGSF3 in the aforementioned pathways remains relatively limited, the findings strongly suggest that these hub genes might affect the overall metabolic balance of sphingolipids by regulating their synthesis, degradation, and signal transduction processes, ultimately influencing the course of AD. By using a systematic bioinformatics approach, we have deciphered the hierarchical control mechanism of key regulatory genes (like SPTLC2, AMD1, and IGSF3) in the pathology of AD. The diagnostic efficacy of these genes has been clinically validated by AUC values, demonstrating significant case differentiation ability. These genes possess dual translational medical value: they serve not only as biomarkers for dynamic disease monitoring but also as pathway-oriented therapeutic targets. The management paradigm of inflammatory skin diseases, including AD, is being transformed by integrating molecular stratification detection, progress tracking, and precise intervention development.

Histopathological evaluation revealed a significant increase in the infiltration of Th2-polarized lymphocytes in AD lesion tissues, accompanied by enhanced immune network interactions. The AMD1/SPTLC2/IGSF3 gene cluster may mediate the inflammatory microenvironment remodeling through hyperactivation of the IL-4/IL-13 pathway, lipid metabolism reprogramming, and transcriptional dysregulation of epidermal barrier proteins. This forms the pathological mechanism of AD, described as “genetic regulation-immune imbalance-tissue damage,” providing a theoretical framework for individualized therapy targeting immune metabolism. Copper-induced cell death, a type of programmed necrosis, is more immunogenic than apoptosis. It triggers inflammatory cytokine release and damage-associated molecular patterns, promoting a proinflammatory state in tissues [48]. Given that AD is a typical type 2 inflammatory skin disease, peripheral blood immune cell activation is closely related to disease severity [49], with Th2 cell polarization dominant [50]. These Th2 cytokines are not only involved in maintaining skin barrier homeostasis [51] but also mediate immune activation and pathological epidermal remodeling through the IL-4Rα/STAT6-dependent signaling network [52]. More importantly, they can induce itchy symptoms in AD by directly activating the primary sensory neurons [53]. Synthesizing these findings, we can speculate that the specific necrotic signaling pathway of cuproptosis may be involved in multiple aspects of AD progression by activating immune cells such as Th2 and producing related proinflammatory cytokines. The existence of persistent skin inflammation in AD may be explained by this complex interaction. Future research should further explore the direct link between cuproptosis and Th2 cell polarization, and how this connection affects the pathogenesis and treatment outcomes of AD.

ROC curve analysis confirmed SPTLC2, AMD1, and IGSF3 with AUC values of 0.804, 0.769, and 0.768, respectively. This multicenter ROC analysis validated SPTLC2 (AUC = 0.804), AMD1 (AUC = 0.769), and IGSF3 (AUC = 0.768) as effective diagnostic biomarkers for distinguishing AD subtypes (95% CIs ≥ 0.75), while establishing clinical-grade cutoff values targeting immune-metabolic pathway abnormalities to enable translational research standards for targeted interventions. The elevated expression levels of SPTLC2, AMD1, and IGSF3 in AD may be linked to their involvement in lipid metabolism and cell signaling-key processes in the pathophysiology of the disease. Expanding the sample size to confirm the diagnostic accuracy of these genes will bolster their credibility as AD biomarkers. Additionally, investigating the expression differences of these genes across various AD subtypes and their clinical significance will deepen our understanding of their specific mechanisms in the disease. Evaluating these genes as targets for pathological testing and drug development could offer novel insights and strategies for precision medicine in AD.

There are some limitations in this study, particularly the issue of inconsistent hub gene levels across different population samples. This may be influenced by various variables such as the choice of measurement method, sample acquisition method, drug treatment, and disease severity. Meanwhile, the existing clinical information is still incomplete and requires validation through multiple methods at the genetic level of clinical samples. To objectively evaluate the effectiveness of SPTLC2, AMD1, and IGSF3 in the diagnosis of AD, subsequent studies need to further verify the applied value of these findings through more experiments and clinical research. Finally, the samples sourced from the database were categorized based on the groupings established by the original authors and could not be well-matched with the clinical subtypes of AD patients. This limitation, to some extent, restricts the generalizability of our conclusions.

## 5. Conclusions

Through systematic computational analysis, this study untangles the core molecular network (SPTLC2, AMD1, IGSF3) and the key metal metabolic regulatory axis (copper homeostasis pathway) involved in the pathophysiology of atopic dermatitis (AD). The findings confirm their potential as diagnostic markers and pathological regulatory functions, providing targeted strategies for precise intervention. The study advocates for multi-dimensional validation and functional research to facilitate clinical translation and application.

## Data Availability

All data generated or analyzed during this study are included in this published article.

## Supplementary Materials

The following supporting information can be downloaded at: https://www.mdpi.com/article/10.3390/biomedicines13061349/s1, Figure S1: Principal component analysis. Table S1: List of 117 genes in the greenyellow module. Table S2: Differency-expressed genes of atopic dermatitis. Table S3: GSEA of atopic dermatitis. Table S4: GO enrichment pathway. Table S5: GeneMania GO enrichment pathway. Table S6: GeneMania KEGG enrichment pathway.
