# Supplementary material for: Decoding Cuproptosis-Sphingolipid-Immune Crosstalk in Atopic Dermatitis: A Multi-Omics Network Analysis
Source: Biomedicines. 2025 May 31;13(6):1349. doi: 10.3390/biomedicines13061349 (PMC12190641; doi:10.3390/biomedicines13061349)
Supplement: Supplementary file 1 [file biomedicines-13-01349-s001.zip › biomedicines-3613116-supplementary.pdf]

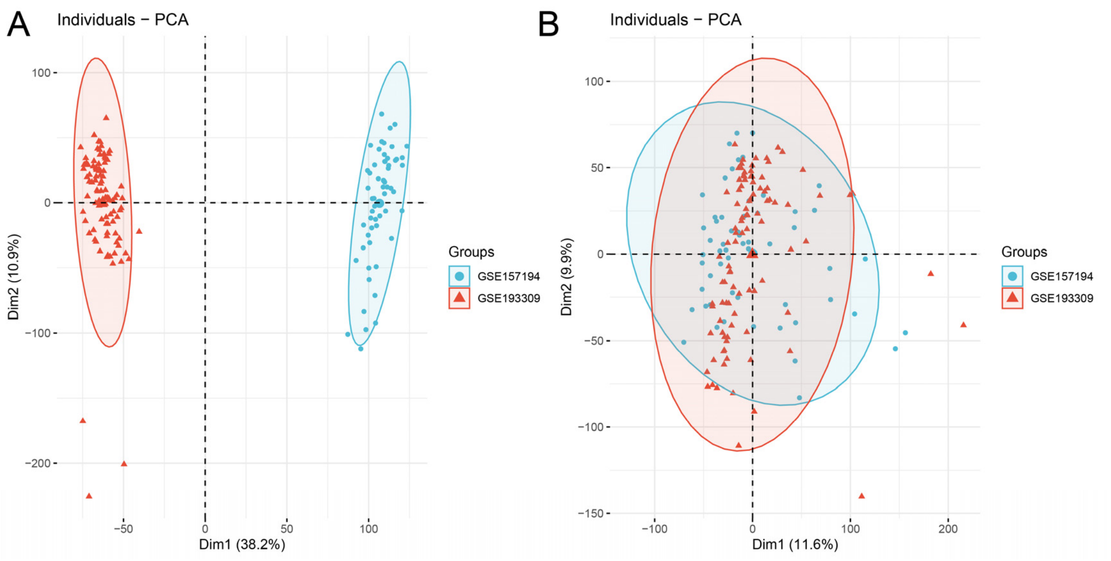

Figure S1. Principal component analysis.

Table S1. List of 117 genes in the greenyellow module

greenyellow_module_genes
CERS2
SORL1
CDC42SE1
LIPN
IGSF3
PRMT3
MTF1
PRPF38A
ITGA2
SPTLC2
UTP11
COPS2
WDR3
DLEU1
MRPS5
CCDC121
BSPRY
ZNF766
ETFA
KCNJ2
TNNI3
CCSAP
FAM83B
DESI2
LRRC57
MYCBP

TMEM8B
ZNF217
TRIM62
CCDC15
POLM
LTN1
TMEM106B
EP300
GDAP1
KAZALD1
NUP155
AMD1
AKAP11
PEX11G
PIK3CA
SASS6
CALU
FNDC3A
ADGRG6
CILK1
KCTD6
ZNF185
CAV2
IPP
SENP7
DIXDC1
FBXO4
ERC1
SATB1
UBE2D1
SLC25A43
TGFB1
PPIL4
LMBRD2
STN1
KIAA1586
AGPAT5
BDP1
NKTR
JARID2
PIGO
ZNF84
C20orf203
ESRP2

STX3
RESF1
MOSPD1
ZBTB39
MCM9
FBXO24
SPART
ZFR
OXER1
PLEKHG3
MGMT
SAAL1
RAPGEF5
PIGL
PAK2
PTDSS2
FSTL3
GAREM1
SECISBP2L
PPIP5K2
CLN6
NFASC
SMCHD1
MOSPD2
RAB30
NUDT15
DNAJA1
POLA1
GAB1
ZNF407
C19orf18
RAPGEF2
CHD7
PM20D2
PPRC1
DOP1A
FAT2
MOCS2
EMILIN1
TTC9C
RALGAPA2
SHPK
ASPSCR1
COPRS

SOCS4
SLC37A3
GAL3ST3

Table S2. Differency-expressed genes of atopic dermatitis

Gene symbol	logFC	*t*	*P*	P_{adj}	β	Group
LCE3E	3.2428	10.8888	＜0.0001	＜0.0001	36.6965	up
LCE3D	3.3530	10.4972	＜0.0001	＜0.0001	34.3379	up
SERPINB4	5.4574	10.4844	＜0.0001	＜0.0001	34.2609	up
S100A7	4.3428	10.4536	＜0.0001	＜0.0001	34.0762	up
LCE3A	4.0910	10.3303	＜0.0001	＜0.0001	33.3362	up
S100A9	4.5878	10.1137	＜0.0001	＜0.0001	32.0407	up
SPRR2B	3.5632	10.0547	＜0.0001	＜0.0001	31.6886	up
RAB3B	-1.7656	-10.0064	＜0.0001	＜0.0001	31.4009	down
BTC	-2.8871	-9.8336	＜0.0001	＜0.0001	30.3729	down
KRT16	3.4877	9.8248	＜0.0001	＜0.0001	30.3208	up
SERPINB3	2.6646	9.6779	＜0.0001	＜0.0001	29.4496	up
SPRR2A	3.8679	9.5753	＜0.0001	＜0.0001	28.8433	up
S100A8	4.3546	9.5393	＜0.0001	＜0.0001	28.6314	up
LINC02687	1.4964	9.5008	＜0.0001	＜0.0001	28.4041	up
AKR1B10	2.3457	9.4609	＜0.0001	＜0.0001	28.1693	up
RETREG1	-1.2274	-9.4553	＜0.0001	＜0.0001	28.1363	down
FUT3	2.0239	9.3944	＜0.0001	＜0.0001	27.7781	up
SPRR1B	2.2147	9.3865	＜0.0001	＜0.0001	27.7317	up
LINC01254	-1.0597	-9.3267	＜0.0001	＜0.0001	27.3806	down
PRSS27	2.1741	9.3236	＜0.0001	＜0.0001	27.3628	up
TMEM45B	1.0004	9.2908	＜0.0001	＜0.0001	27.1703	up
CLCN1	1.3467	9.2709	＜0.0001	＜0.0001	27.0539	up
C10orf99	2.1668	9.2609	＜0.0001	＜0.0001	26.9952	up
KRT6C	4.2701	9.2542	＜0.0001	＜0.0001	26.9564	up
ZDHHC9	-0.8140	-9.2218	＜0.0001	＜0.0001	26.7668	down
UGT3A2	-1.7259	-9.1993	＜0.0001	＜0.0001	26.6353	down
PPP4R1	0.7165	9.1965	＜0.0001	＜0.0001	26.6190	up
AGBL4	-1.1897	-9.1788	＜0.0001	＜0.0001	26.5155	down
CLEC7A	1.5260	9.0591	＜0.0001	＜0.0001	25.8177	up
CCND1	-0.8725	-8.9861	＜0.0001	＜0.0001	25.3938	down
ABCG4	1.7164	8.9464	＜0.0001	＜0.0001	25.1633	up
CHRM4	-1.9618	-8.9285	＜0.0001	＜0.0001	25.0599	down
PAK3	-0.9342	-8.8833	＜0.0001	＜0.0001	24.7983	down
SPRR2G	2.8812	8.8740	＜0.0001	＜0.0001	24.7443	up
MTR	-0.5649	-8.8730	＜0.0001	＜0.0001	24.7385	down
QSOX1	0.8149	8.7254	＜0.0001	＜0.0001	23.8877	up
ZNF429	-0.6608	-8.7208	＜0.0001	＜0.0001	23.8616	down

SPRR2D	2.6123	8.6684	＜0.0001	＜0.0001	23.5605	up
POLR3G	1.2168	8.6355	＜0.0001	＜0.0001	23.3718	up
GALNT6	1.1432	8.5789	＜0.0001	＜0.0001	23.0477	up
SPRR1A	2.3707	8.5719	＜0.0001	＜0.0001	23.0077	up
ANKRD36C	-0.7656	-8.5155	＜0.0001	＜0.0001	22.6858	down
GJB2	1.9967	8.4873	＜0.0001	＜0.0001	22.5248	up
IL36G	1.5229	8.4623	＜0.0001	＜0.0001	22.3828	up
RAI14	-0.8885	-8.4529	＜0.0001	＜0.0001	22.3291	down
KRT77	-1.7030	-8.4430	＜0.0001	＜0.0001	22.2728	down
SERPINB13	1.2817	8.4390	＜0.0001	＜0.0001	22.2501	up
LINC01605	0.9800	8.4347	＜0.0001	＜0.0001	22.2259	up
ZNF418	-0.5791	-8.4212	＜0.0001	＜0.0001	22.1490	down
CACNA1H	-1.3258	-8.4149	＜0.0001	＜0.0001	22.1132	down
S100A7A	3.8962	8.3986	＜0.0001	＜0.0001	22.0209	up
ZSCAN18	-1.0885	-8.3952	＜0.0001	＜0.0001	22.0013	down
NLRX1	0.8171	8.3908	＜0.0001	＜0.0001	21.9765	up
RAB31	1.0369	8.3505	＜0.0001	＜0.0001	21.7479	up
CMC4	-0.8891	-8.3478	＜0.0001	＜0.0001	21.7330	down
GSTM2	-0.9149	-8.3466	＜0.0001	＜0.0001	21.7258	down
LCE5A	-2.6639	-8.3420	＜0.0001	＜0.0001	21.6998	down
FUT2	0.9986	8.3375	＜0.0001	＜0.0001	21.6745	up
SH3KBP1	-0.7484	-8.3286	＜0.0001	＜0.0001	21.6243	down
ADH6	-0.7448	-8.3279	＜0.0001	＜0.0001	21.6198	down
MFHAS1	0.6891	8.3197	＜0.0001	＜0.0001	21.5739	up
SPDYE2	-1.3925	-8.3067	＜0.0001	＜0.0001	21.5000	down
F3	-1.3696	-8.2965	＜0.0001	＜0.0001	21.4425	down
C14orf132	-1.0105	-8.2939	＜0.0001	＜0.0001	21.4281	down
PLA2G2F	0.9652	8.2711	＜0.0001	＜0.0001	21.2990	up
HPSE	1.2117	8.2682	＜0.0001	＜0.0001	21.2829	up
ZNF302	-0.6054	-8.2530	＜0.0001	＜0.0001	21.1972	down
RPL13	-0.5825	-8.2368	＜0.0001	＜0.0001	21.1058	down
PVT1	0.7662	8.2332	＜0.0001	＜0.0001	21.0853	up
FAM234B	-1.1585	-8.2328	＜0.0001	＜0.0001	21.0830	down
TNFRSF21	1.2167	8.1921	＜0.0001	＜0.0001	20.8542	up
DSG3	1.0883	8.1585	＜0.0001	＜0.0001	20.6652	up
SYNE1	-0.8337	-8.1389	＜0.0001	＜0.0001	20.5554	down
FSCN1	0.9684	8.1268	＜0.0001	＜0.0001	20.4872	up
S100A2	1.3208	8.1241	＜0.0001	＜0.0001	20.4725	up
GAN	-1.1772	-8.1195	＜0.0001	＜0.0001	20.4468	down
USP54	-0.6100	-8.1164	＜0.0001	＜0.0001	20.4293	down
FURIN	0.5788	8.1109	＜0.0001	＜0.0001	20.3983	up
RPS19	-0.5750	-8.0873	＜0.0001	＜0.0001	20.2661	down
DNASE1L3	1.1661	8.0799	＜0.0001	＜0.0001	20.2250	up
TCN1	2.7778	8.0592	＜0.0001	＜0.0001	20.1092	up

KCNK10	0.8377	8.0421	＜0.0001	＜0.0001	20.0137	up
DPP4	1.0856	8.0364	＜0.0001	＜0.0001	19.9818	up
RHOBTB3	-0.9293	-8.0281	＜0.0001	＜0.0001	19.9358	down
EPHX2	-0.7700	-8.0152	＜0.0001	＜0.0001	19.8638	down
PHYHIP	-1.6062	-8.0045	＜0.0001	＜0.0001	19.8039	down
IL37	-1.9961	-7.9993	＜0.0001	＜0.0001	19.7749	down
APOE	-1.3961	-7.9821	＜0.0001	＜0.0001	19.6794	down
MAPK6	0.8096	7.9796	＜0.0001	＜0.0001	19.6650	up
SUSD2	-1.4635	-7.9654	＜0.0001	＜0.0001	19.5865	down
TREX2	1.2314	7.9591	＜0.0001	＜0.0001	19.5512	up
FGD2	0.9080	7.9233	＜0.0001	＜0.0001	19.3522	up
CD24	1.1571	7.9228	＜0.0001	＜0.0001	19.3499	up
DSC2	1.4815	7.9201	＜0.0001	＜0.0001	19.3348	up
IFI27	1.9613	7.9122	＜0.0001	＜0.0001	19.2906	up
SCD5	-0.9563	-7.9111	＜0.0001	＜0.0001	19.2847	down
ZNF577	-0.8337	-7.9107	＜0.0001	＜0.0001	19.2825	down
SLC6A11	1.0043	7.9055	＜0.0001	＜0.0001	19.2535	up
CTSC	1.2310	7.8966	＜0.0001	＜0.0001	19.2046	up
XPC	-0.5412	-7.8939	＜0.0001	＜0.0001	19.1896	down
MXD1	0.9873	7.8935	＜0.0001	＜0.0001	19.1870	up
OASL	1.8884	7.8872	＜0.0001	＜0.0001	19.1524	up
EXOC3	0.5159	7.8861	＜0.0001	＜0.0001	19.1460	up
RPS2	-0.5897	-7.8734	＜0.0001	＜0.0001	19.0758	down
SPRR2E	1.7668	7.8656	＜0.0001	＜0.0001	19.0330	up
TPBG	0.8154	7.8540	＜0.0001	＜0.0001	18.9684	up
SCCPDH	-0.9647	-7.8457	＜0.0001	＜0.0001	18.9229	down
OAS3	0.9757	7.8227	＜0.0001	＜0.0001	18.7954	up
HSD11B1	-2.1798	-7.8199	＜0.0001	＜0.0001	18.7804	down
FABP5	1.2376	7.8067	＜0.0001	＜0.0001	18.7074	up
PTPN14	-0.5945	-7.7979	＜0.0001	＜0.0001	18.6588	down
ARNTL2	0.6468	7.7903	＜0.0001	＜0.0001	18.6171	up
C5orf46	-2.1448	-7.7862	＜0.0001	＜0.0001	18.5942	down
TTC39A	1.0643	7.7616	＜0.0001	＜0.0001	18.4588	up
AFAP1L2	0.6857	7.7497	＜0.0001	＜0.0001	18.3934	up
RGS20	1.0288	7.7494	＜0.0001	＜0.0001	18.3917	up
FRMD4A	-0.7113	-7.7412	＜0.0001	＜0.0001	18.3466	down
ZNF135	-0.8763	-7.7338	＜0.0001	＜0.0001	18.3061	down
WIF1	-1.5759	-7.7180	＜0.0001	＜0.0001	18.2195	down
TYMP	1.6403	7.7161	＜0.0001	＜0.0001	18.2088	up
ENO1	0.6399	7.7103	＜0.0001	＜0.0001	18.1771	up
HEPHL1	1.9430	7.6966	＜0.0001	＜0.0001	18.1020	up
SCIN	-1.1157	-7.6828	＜0.0001	＜0.0001	18.0262	down
GJB6	1.4429	7.6780	＜0.0001	＜0.0001	18.0003	up
ALDH3A2	-0.7869	-7.6501	＜0.0001	＜0.0001	17.8476	down

CYP2J2	-1.2811	-7.6496	＜0.0001	＜0.0001	17.8449	down
SPRR2F	3.0320	7.6382	＜0.0001	＜0.0001	17.7825	up
PI3	2.9907	7.6365	＜0.0001	＜0.0001	17.7731	up
KRT6A	2.7702	7.6122	＜0.0001	＜0.0001	17.6407	up
PELATON	1.2651	7.5998	＜0.0001	＜0.0001	17.5730	up
FGD3	0.9250	7.5919	＜0.0001	＜0.0001	17.5296	up
TMPRSS11F	-0.9331	-7.5876	＜0.0001	＜0.0001	17.5063	down
ADAP2	0.7199	7.5870	＜0.0001	＜0.0001	17.5029	up
KIF21A	-0.6415	-7.5619	＜0.0001	＜0.0001	17.3665	down
SCEL	-1.1399	-7.5446	＜0.0001	＜0.0001	17.2725	down
NTM	-0.7315	-7.5266	＜0.0001	＜0.0001	17.1747	down
IVL	0.9660	7.5171	＜0.0001	＜0.0001	17.1232	up
PTPRE	0.7318	7.5123	＜0.0001	＜0.0001	17.0971	up
ASAH1	-0.5147	-7.5017	＜0.0001	＜0.0001	17.0395	down
TMEM43	-0.5458	-7.4891	＜0.0001	＜0.0001	16.9713	down
ARHGEF10	-0.7985	-7.4819	＜0.0001	＜0.0001	16.9326	down
LRRC59	0.7299	7.4480	＜0.0001	＜0.0001	16.7491	up
IFI16	0.5127	7.4453	＜0.0001	＜0.0001	16.7343	up
CYP2C9	-0.9210	-7.4361	＜0.0001	＜0.0001	16.6851	down
PSG5	-0.7232	-7.4344	＜0.0001	＜0.0001	16.6756	down
GLIPR1L1	-1.4950	-7.4305	＜0.0001	＜0.0001	16.6548	down
ANKRD36	-0.5940	-7.4249	＜0.0001	＜0.0001	16.6246	down
EPHA1	0.6839	7.4217	＜0.0001	＜0.0001	16.6069	up
TCAF2	0.7563	7.4160	＜0.0001	＜0.0001	16.5764	up
TGFBI	-0.7371	-7.4156	＜0.0001	＜0.0001	16.5745	down
CDH12	-0.5716	-7.4111	＜0.0001	＜0.0001	16.5502	down
MDFI	1.1200	7.4100	＜0.0001	＜0.0001	16.5440	up
PNP	0.8794	7.4035	＜0.0001	＜0.0001	16.5093	up
KMT2E	-0.6519	-7.3959	＜0.0001	＜0.0001	16.4681	down
ACP5	1.0089	7.3700	＜0.0001	＜0.0001	16.3286	up
GPLD1	-1.0575	-7.3692	＜0.0001	＜0.0001	16.3247	down
CHAD	-0.8630	-7.3608	＜0.0001	＜0.0001	16.2793	down
FHOD3	-0.6173	-7.3583	＜0.0001	＜0.0001	16.2658	down
SERPINA12	-1.2425	-7.3473	＜0.0001	＜0.0001	16.2070	down
ZNF493	-0.5401	-7.3323	＜0.0001	＜0.0001	16.1264	down
TSPYL2	-0.7270	-7.3207	＜0.0001	＜0.0001	16.0643	down
EIF4EBP1	0.8059	7.3152	＜0.0001	＜0.0001	16.0345	up
VSNL1	0.9058	7.3140	＜0.0001	＜0.0001	16.0282	up
CLDN23	-1.4733	-7.3046	＜0.0001	＜0.0001	15.9782	down
CNFN	0.7772	7.3013	＜0.0001	＜0.0001	15.9603	up
PRKCB	-0.8559	-7.2960	＜0.0001	＜0.0001	15.9322	down
ZNF540	-0.5042	-7.2946	＜0.0001	＜0.0001	15.9244	down
NBPF1	-0.5938	-7.2944	＜0.0001	＜0.0001	15.9237	down
MIR99AHG	-0.5831	-7.2944	＜0.0001	＜0.0001	15.9235	down

RRM2	1.1740	7.2890	＜0.0001	＜0.0001	15.8945	up
ZNF439	-0.6495	-7.2878	＜0.0001	＜0.0001	15.8882	down
IL34	-1.1757	-7.2739	＜0.0001	＜0.0001	15.8139	down
IRF7	0.8876	7.2719	＜0.0001	＜0.0001	15.8030	up
PLA2G4D	1.1722	7.2604	＜0.0001	＜0.0001	15.7421	up
RAET1G	0.9015	7.2566	＜0.0001	＜0.0001	15.7213	up
CFAP251	1.3479	7.2550	＜0.0001	＜0.0001	15.7130	up
USB1	0.7992	7.2545	＜0.0001	＜0.0001	15.7102	up
CYP2W1	-0.7078	-7.2530	＜0.0001	＜0.0001	15.7021	down
CRABP1	-1.2950	-7.2406	＜0.0001	＜0.0001	15.6362	down
RASSF6	-0.5486	-7.2369	＜0.0001	＜0.0001	15.6166	down
FMO2	1.6531	7.2319	＜0.0001	＜0.0001	15.5896	up
PRR11	0.8300	7.2011	＜0.0001	＜0.0001	15.4259	up
RGMB	-0.6395	-7.2004	＜0.0001	＜0.0001	15.4223	down
DEFB4A	2.3660	7.1991	＜0.0001	＜0.0001	15.4154	up
IMPDH1	0.5718	7.1951	＜0.0001	＜0.0001	15.3940	up
GDA	1.3557	7.1923	＜0.0001	＜0.0001	15.3790	up
PCLAF	0.8849	7.1843	＜0.0001	＜0.0001	15.3368	up
C2CD2	-0.7273	-7.1831	＜0.0001	＜0.0001	15.3305	down
TXNIP	-0.7109	-7.1780	＜0.0001	＜0.0001	15.3030	down
ZNF573	-0.5764	-7.1647	＜0.0001	＜0.0001	15.2325	down
CDC25B	0.8406	7.1570	＜0.0001	＜0.0001	15.1920	up
ZC3H6	-0.5264	-7.1496	＜0.0001	＜0.0001	15.1528	down
ASH1L	-0.5789	-7.1434	＜0.0001	＜0.0001	15.1200	down
SMIM18	-1.2778	-7.1429	＜0.0001	＜0.0001	15.1170	down
MARCHF6	-0.6066	-7.1420	＜0.0001	＜0.0001	15.1124	down
LINC02747	-0.6546	-7.1416	＜0.0001	＜0.0001	15.1103	down
WNT2B	-0.7830	-7.1383	＜0.0001	＜0.0001	15.0931	down
LINC02232	-0.6078	-7.1119	＜0.0001	＜0.0001	14.9532	down
AKR1B15	1.2492	7.1065	＜0.0001	＜0.0001	14.9249	up
MPHOSPH6	0.5832	7.1003	＜0.0001	＜0.0001	14.8918	up
ERO1A	0.6202	7.0987	＜0.0001	＜0.0001	14.8834	up
CYP39A1	-0.8980	-7.0986	＜0.0001	＜0.0001	14.8828	down
SPDYE2B	-1.1444	-7.0970	＜0.0001	＜0.0001	14.8743	down
RPS6KA4	0.7490	7.0930	＜0.0001	＜0.0001	14.8532	up
GALNT1	-0.5633	-7.0925	＜0.0001	＜0.0001	14.8509	down
ZNF606	-0.5547	-7.0846	＜0.0001	＜0.0001	14.8093	down
TMPRSS4	1.5031	7.0839	＜0.0001	＜0.0001	14.8053	up
KRT15	-1.6782	-7.0828	＜0.0001	＜0.0001	14.7994	down
TMEM165	0.5510	7.0802	＜0.0001	＜0.0001	14.7858	up
ZNF106	-0.5294	-7.0795	＜0.0001	＜0.0001	14.7821	down
SNTB1	-0.8849	-7.0662	＜0.0001	＜0.0001	14.7124	down
SVIP	-0.7120	-7.0453	＜0.0001	＜0.0001	14.6021	down
ACADL	-0.6540	-7.0343	＜0.0001	＜0.0001	14.5445	down

RNPC3	-0.7260	-7.0009	＜0.0001	＜0.0001	14.3692	down
ZNF528	-0.5037	-6.9901	＜0.0001	＜0.0001	14.3124	down
RUFY3	-0.5032	-6.9777	＜0.0001	＜0.0001	14.2475	down
CYCS	0.5364	6.9701	＜0.0001	＜0.0001	14.2078	up
LGALS9B	0.5179	6.9615	＜0.0001	＜0.0001	14.1630	up
AGR3	-1.2210	-6.9597	＜0.0001	＜0.0001	14.1532	down
IL4R	1.0610	6.9596	＜0.0001	＜0.0001	14.1531	up
EHF	1.1108	6.9575	＜0.0001	＜0.0001	14.1420	up
ZNF559	-0.6918	-6.9564	＜0.0001	＜0.0001	14.1363	down
PLLP	-1.0653	-6.9466	＜0.0001	＜0.0001	14.0848	down
PAIP2B	-0.6614	-6.9451	＜0.0001	＜0.0001	14.0771	down
TNIP2	0.5223	6.9230	＜0.0001	＜0.0001	13.9618	up
SGK1	0.7536	6.9220	＜0.0001	＜0.0001	13.9565	up
CCL18	2.5009	6.9052	＜0.0001	＜0.0001	13.8691	up
CXCL14	-0.7687	-6.9041	＜0.0001	＜0.0001	13.8637	down
MX1	1.2359	6.9014	＜0.0001	＜0.0001	13.8496	up
LIPH	-0.8818	-6.8988	＜0.0001	＜0.0001	13.8357	down
FOSL1	1.5623	6.8902	＜0.0001	＜0.0001	13.7913	up
PLXNA4	-0.6894	-6.8721	＜0.0001	＜0.0001	13.6970	down
NUAK1	-0.8972	-6.8710	＜0.0001	＜0.0001	13.6912	down
RALGPS2	0.7285	6.8647	＜0.0001	＜0.0001	13.6585	up
LINC01659	-0.7583	-6.8577	＜0.0001	＜0.0001	13.6223	down
ZNF83	-0.5703	-6.8551	＜0.0001	＜0.0001	13.6091	down
ADD3	-0.5770	-6.8487	＜0.0001	＜0.0001	13.5758	down
CACNB2	-0.5213	-6.8460	＜0.0001	＜0.0001	13.5620	down
NELL2	1.4064	6.8305	＜0.0001	＜0.0001	13.4818	up
KRTDAP	-0.6437	-6.8303	＜0.0001	＜0.0001	13.4803	down
C10orf67	-0.8306	-6.8223	＜0.0001	＜0.0001	13.4390	down
ID4	-1.1908	-6.8223	＜0.0001	＜0.0001	13.4389	down
OSMR	0.6287	6.8189	＜0.0001	＜0.0001	13.4215	up
IL36RN	0.7883	6.8188	＜0.0001	＜0.0001	13.4211	up
ABCA5	-0.5480	-6.8131	＜0.0001	＜0.0001	13.3918	down
TGM1	0.8631	6.8123	＜0.0001	＜0.0001	13.3874	up
ZFP14	-0.5492	-6.7987	＜0.0001	＜0.0001	13.3170	down
ERBB4	-0.6253	-6.7967	＜0.0001	＜0.0001	13.3067	down
GM2A	0.5533	6.7959	＜0.0001	＜0.0001	13.3029	up
ZBTB16	-1.2410	-6.7916	＜0.0001	＜0.0001	13.2806	down
ELF3	0.8868	6.7900	＜0.0001	＜0.0001	13.2721	up
RDH16	0.9624	6.7880	＜0.0001	＜0.0001	13.2621	up
PYGL	0.7401	6.7799	＜0.0001	＜0.0001	13.2200	up
N4BP2L1	-0.6315	-6.7778	＜0.0001	＜0.0001	13.2094	down
C1orf68	-1.0895	-6.7746	＜0.0001	＜0.0001	13.1928	down
PKMYT1	0.8683	6.7705	＜0.0001	＜0.0001	13.1720	up
AIF1L	-0.6644	-6.7697	＜0.0001	＜0.0001	13.1677	down

BCL6	-0.7828	-6.7692	＜0.0001	＜0.0001	13.1652	down
LCN2	1.8591	6.7615	＜0.0001	＜0.0001	13.1252	up
PLK1	0.7744	6.7560	＜0.0001	＜0.0001	13.0969	up
CHAC1	1.7057	6.7557	＜0.0001	＜0.0001	13.0957	up
SLC52A3	0.8321	6.7435	＜0.0001	＜0.0001	13.0326	up
ANKRD18A	-0.7635	-6.7326	＜0.0001	＜0.0001	12.9771	down
GSDME	0.9705	6.7253	＜0.0001	＜0.0001	12.9395	up
TREML1	0.7974	6.7098	＜0.0001	＜0.0001	12.8600	up
CD36	1.3874	6.7076	＜0.0001	＜0.0001	12.8484	up
LINC02345	-0.7800	-6.7044	＜0.0001	＜0.0001	12.8323	down
WNT2	-0.6496	-6.7020	＜0.0001	＜0.0001	12.8199	down
NAPEPLD	-0.5777	-6.6980	＜0.0001	＜0.0001	12.7991	down
SPINK1	-0.6040	-6.6958	＜0.0001	＜0.0001	12.7881	down
SOX7	0.7403	6.6885	＜0.0001	＜0.0001	12.7506	up
KLRF2	-0.5591	-6.6883	＜0.0001	＜0.0001	12.7497	down
PPP3CA	-0.5223	-6.6863	＜0.0001	＜0.0001	12.7397	down
MPZL2	0.9587	6.6803	＜0.0001	＜0.0001	12.7087	up
RIC3	-0.6804	-6.6783	＜0.0001	＜0.0001	12.6984	down
FAM227A	-0.7852	-6.6690	＜0.0001	＜0.0001	12.6511	down
INA	0.7702	6.6647	＜0.0001	＜0.0001	12.6290	up
CLDN16	-0.6426	-6.6597	＜0.0001	＜0.0001	12.6035	down
BIRC5	0.8912	6.6571	＜0.0001	＜0.0001	12.5903	up
CENPE	0.5599	6.6504	＜0.0001	＜0.0001	12.5560	up
AQP9	-0.9710	-6.6491	＜0.0001	＜0.0001	12.5494	down
LUC7L3	-0.5629	-6.6347	＜0.0001	＜0.0001	12.4762	down
CFAP57	-0.6868	-6.6318	＜0.0001	＜0.0001	12.4614	down
BCAR3	-0.8771	-6.6225	＜0.0001	＜0.0001	12.4137	down
ACKR2	1.3732	6.6167	＜0.0001	＜0.0001	12.3842	up
TSGA10	-0.5359	-6.6078	＜0.0001	＜0.0001	12.3392	down
PPARD	0.8785	6.6024	＜0.0001	＜0.0001	12.3118	up
LRP4	-0.8187	-6.5925	＜0.0001	＜0.0001	12.2613	down
TNC	1.3095	6.5865	＜0.0001	＜0.0001	12.2308	up
CDKN1B	-0.5979	-6.5832	＜0.0001	＜0.0001	12.2142	down
NOD2	0.7263	6.5786	＜0.0001	＜0.0001	12.1910	up
SLAMF7	0.6520	6.5742	＜0.0001	＜0.0001	12.1688	up
NAMPT	0.8372	6.5732	＜0.0001	＜0.0001	12.1636	up
JAK3	0.9798	6.5681	＜0.0001	＜0.0001	12.1376	up
LIF	-0.6218	-6.5653	＜0.0001	＜0.0001	12.1236	down
TMEM80	-0.5084	-6.5565	＜0.0001	＜0.0001	12.0787	down
MOXD1	0.8241	6.5552	＜0.0001	＜0.0001	12.0722	up
MIR181A2HG	-0.6449	-6.5474	＜0.0001	＜0.0001	12.0327	down
FAT1	0.7846	6.5351	＜0.0001	＜0.0001	11.9705	up
CLDN8	-0.7754	-6.5348	＜0.0001	＜0.0001	11.9692	down
COL21A1	-0.6276	-6.5333	＜0.0001	＜0.0001	11.9617	down

B3GNT3	0.9015	6.5320	＜0.0001	＜0.0001	11.9553	up
CARD6	0.8694	6.5301	＜0.0001	＜0.0001	11.9457	up
C7orf57	1.2218	6.5197	＜0.0001	＜0.0001	11.8930	up
GGH	0.7664	6.5166	＜0.0001	＜0.0001	11.8774	up
TTC38	-0.9346	-6.5161	＜0.0001	＜0.0001	11.8749	down
ZNF420	-0.5430	-6.5134	＜0.0001	＜0.0001	11.8613	down
KCNK6	0.5003	6.5086	＜0.0001	＜0.0001	11.8370	up
NOP53	-0.7897	-6.5081	＜0.0001	＜0.0001	11.8346	down
CCL24	1.7925	6.5058	＜0.0001	＜0.0001	11.8228	up
CHI3L2	1.8454	6.5030	＜0.0001	＜0.0001	11.8087	up
SLC5A1	0.6908	6.5026	＜0.0001	＜0.0001	11.8065	up
EEF2K	-0.6621	-6.4961	＜0.0001	＜0.0001	11.7738	down
PLAGL1	-0.5569	-6.4936	＜0.0001	＜0.0001	11.7615	down
RORC	-0.7635	-6.4905	＜0.0001	＜0.0001	11.7456	down
GCOM1	0.8594	6.4860	＜0.0001	＜0.0001	11.7229	up
SLC26A5	-0.7265	-6.4843	＜0.0001	＜0.0001	11.7145	down
FCHSD1	0.7703	6.4820	＜0.0001	＜0.0001	11.7031	up
LORICRIN	-1.5243	-6.4810	＜0.0001	＜0.0001	11.6982	down
STUM	-0.8843	-6.4810	＜0.0001	＜0.0001	11.6982	down
TMEM255A	-0.5692	-6.4785	＜0.0001	＜0.0001	11.6853	down
SOX5	-0.7858	-6.4773	＜0.0001	＜0.0001	11.6792	down
SMOX	1.0800	6.4622	＜0.0001	＜0.0001	11.6038	up
BBS2	-0.5813	-6.4590	＜0.0001	＜0.0001	11.5875	down
STRIP2	0.6429	6.4585	＜0.0001	＜0.0001	11.5848	up
GSX2	-1.5420	-6.4576	＜0.0001	＜0.0001	11.5803	down
SHCBP1	0.6606	6.4572	＜0.0001	＜0.0001	11.5787	up
PLAC1	-0.7070	-6.4559	＜0.0001	＜0.0001	11.5717	down
CYP7B1	0.5877	6.4500	＜0.0001	＜0.0001	11.5421	up
CCNB1	0.9201	6.4474	＜0.0001	＜0.0001	11.5293	up
PGM2	0.5720	6.4413	＜0.0001	＜0.0001	11.4985	up
FLG2	-1.2237	-6.4405	＜0.0001	＜0.0001	11.4945	down
ZNF415	-0.5668	-6.4390	＜0.0001	＜0.0001	11.4874	down
HOPX	-0.6187	-6.4381	＜0.0001	＜0.0001	11.4827	down
FBXO45	0.6131	6.4362	＜0.0001	＜0.0001	11.4730	up
CHP2	-1.0836	-6.4322	＜0.0001	＜0.0001	11.4529	down
PITX1	1.1221	6.4296	＜0.0001	＜0.0001	11.4402	up
HTR3A	1.4134	6.4295	＜0.0001	＜0.0001	11.4396	up
PITPNM1	0.5759	6.4260	＜0.0001	＜0.0001	11.4219	up
C5AR2	0.5361	6.4213	＜0.0001	＜0.0001	11.3987	up
F12	0.8069	6.4190	＜0.0001	＜0.0001	11.3869	up
PTTG1	0.7382	6.4189	＜0.0001	＜0.0001	11.3866	up
SLC7A11	0.8493	6.4087	＜0.0001	＜0.0001	11.3354	up
ZNF717	-0.5222	-6.4068	＜0.0001	＜0.0001	11.3261	down
ANKRD12	-0.5048	-6.4017	＜0.0001	＜0.0001	11.3007	down

TP53I3	0.6700	6.3996	＜0.0001	＜0.0001	11.2899	up
SPIN4	0.8149	6.3942	＜0.0001	＜0.0001	11.2632	up
ZNF626	-0.5051	-6.3931	＜0.0001	＜0.0001	11.2577	down
AMMECR1	0.5290	6.3895	＜0.0001	＜0.0001	11.2398	up
CES4A	-0.6854	-6.3846	＜0.0001	＜0.0001	11.2152	down
ARHGEF26	-0.6508	-6.3845	＜0.0001	＜0.0001	11.2149	down
VSIG10	-0.5643	-6.3838	＜0.0001	＜0.0001	11.2115	down
PHYHD1	-0.7507	-6.3756	＜0.0001	＜0.0001	11.1703	down
CXCL17	0.7534	6.3736	＜0.0001	＜0.0001	11.1605	up
UGT1A7	1.5407	6.3653	＜0.0001	＜0.0001	11.1191	up
DCUN1D3	0.5180	6.3555	＜0.0001	＜0.0001	11.0703	up
SYT17	-0.5628	-6.3514	＜0.0001	＜0.0001	11.0500	down
OGDHL	1.2824	6.3502	＜0.0001	＜0.0001	11.0439	up
LNC-LBCS	-0.5672	-6.3465	＜0.0001	＜0.0001	11.0255	down
RAPGEF4	-0.5724	-6.3449	＜0.0001	＜0.0001	11.0176	down
RXRA	-0.5611	-6.3446	＜0.0001	＜0.0001	11.0164	down
IL2RB	-0.5815	-6.3445	＜0.0001	＜0.0001	11.0160	down
MYBL2	1.0036	6.3436	＜0.0001	＜0.0001	11.0110	up
HS3ST6	-1.2163	-6.3415	＜0.0001	＜0.0001	11.0009	down
ZNF677	-0.5622	-6.3364	＜0.0001	＜0.0001	10.9756	down
MLXIP	-0.5020	-6.3328	＜0.0001	＜0.0001	10.9575	down
ORMDL1	-0.5299	-6.3254	＜0.0001	＜0.0001	10.9212	down
ANKRD33B	-0.8604	-6.3251	＜0.0001	＜0.0001	10.9196	down
ARL5B	0.5538	6.3194	＜0.0001	＜0.0001	10.8911	up
SLC4A7	0.8144	6.3194	＜0.0001	＜0.0001	10.8910	up
OAS1	0.8686	6.3129	＜0.0001	＜0.0001	10.8591	up
HRH1	0.8497	6.3125	＜0.0001	＜0.0001	10.8569	up
HMOX2	0.5397	6.3064	＜0.0001	＜0.0001	10.8266	up
FAM13C	-0.5003	-6.3000	＜0.0001	＜0.0001	10.7953	down
CDC42EP1	0.5908	6.2995	＜0.0001	＜0.0001	10.7925	up
WNK2	-0.7803	-6.2989	＜0.0001	＜0.0001	10.7896	down
LRRC20	0.6431	6.2975	＜0.0001	＜0.0001	10.7829	up
CEACAM19	0.5880	6.2950	＜0.0001	＜0.0001	10.7702	up
TMPRSS6	-0.9194	-6.2935	＜0.0001	＜0.0001	10.7631	down
CEP55	0.6262	6.2930	＜0.0001	＜0.0001	10.7605	up
CH25H	0.9410	6.2913	＜0.0001	＜0.0001	10.7522	up
TPRG1	-0.5221	-6.2799	＜0.0001	＜0.0001	10.6955	down
ANGPTL4	1.2890	6.2784	＜0.0001	＜0.0001	10.6882	up
TK1	0.7786	6.2753	＜0.0001	＜0.0001	10.6729	up
PLCD4	0.7109	6.2724	＜0.0001	＜0.0001	10.6586	up
NPIPB6	-0.7902	-6.2722	＜0.0001	＜0.0001	10.6577	down
TNFRSF25	-0.6855	-6.2698	＜0.0001	＜0.0001	10.6460	down
ZFP36	0.6605	6.2692	＜0.0001	＜0.0001	10.6431	up
DZIP1L	-0.5374	-6.2624	＜0.0001	＜0.0001	10.6093	down

SELE	1.4653	6.2590	＜0.0001	＜0.0001	10.5925	up
EDIL3	-0.6882	-6.2559	＜0.0001	＜0.0001	10.5776	down
TNFSF10	0.6341	6.2553	＜0.0001	＜0.0001	10.5745	up
SLC7A5	0.7801	6.2515	＜0.0001	＜0.0001	10.5556	up
IRS2	-0.6776	-6.2474	＜0.0001	＜0.0001	10.5357	down
NEIL1	-0.5871	-6.2435	＜0.0001	＜0.0001	10.5163	down
TIGAR	0.5947	6.2423	＜0.0001	＜0.0001	10.5102	up
APOBEC3B	0.8630	6.2414	＜0.0001	＜0.0001	10.5059	up
CD47	0.5696	6.2386	＜0.0001	＜0.0001	10.4920	up
CCDC3	-0.7081	-6.2374	＜0.0001	＜0.0001	10.4864	down
GPRIN1	0.7097	6.2356	＜0.0001	＜0.0001	10.4774	up
WFS1	-0.5876	-6.2321	＜0.0001	＜0.0001	10.4604	down
CLEC2A	-1.2232	-6.2316	＜0.0001	＜0.0001	10.4580	down
RIPOR3	0.8000	6.2296	＜0.0001	＜0.0001	10.4479	up
IL12RB2	0.7217	6.2295	＜0.0001	＜0.0001	10.4474	up
SLC16A1	0.6382	6.2290	＜0.0001	＜0.0001	10.4451	up
SPAG1	-0.6083	-6.2290	＜0.0001	＜0.0001	10.4450	down
SFN	0.6203	6.2259	＜0.0001	＜0.0001	10.4299	up
FAM166B	-0.5195	-6.2210	＜0.0001	＜0.0001	10.4058	down
CCND2	0.5723	6.2192	＜0.0001	＜0.0001	10.3968	up
MACROD2	-0.5896	-6.2167	＜0.0001	＜0.0001	10.3848	down
GATA3	-0.7878	-6.2131	＜0.0001	＜0.0001	10.3668	down
TRIM10	0.9604	6.2056	＜0.0001	＜0.0001	10.3301	up
TUBA1C	0.6084	6.2026	＜0.0001	＜0.0001	10.3155	up
KPNA2	0.8858	6.2022	＜0.0001	＜0.0001	10.3134	up
WFDC12	1.0543	6.2003	＜0.0001	＜0.0001	10.3040	up
UNC13D	0.5783	6.1980	＜0.0001	＜0.0001	10.2927	up
MYD88	0.5070	6.1965	＜0.0001	＜0.0001	10.2853	up
GPC2	-0.5032	-6.1961	＜0.0001	＜0.0001	10.2834	down
TRAPPC6A	-0.6648	-6.1871	＜0.0001	＜0.0001	10.2394	down
ABHD12B	-1.0555	-6.1849	＜0.0001	＜0.0001	10.2286	down
LY6D	0.5407	6.1826	＜0.0001	＜0.0001	10.2172	up
MMP12	2.2727	6.1794	＜0.0001	＜0.0001	10.2019	up
AUNIP	0.7986	6.1762	＜0.0001	＜0.0001	10.1860	up
FBXW7	-0.5496	-6.1745	＜0.0001	＜0.0001	10.1777	down
LFNG	-0.6339	-6.1688	＜0.0001	＜0.0001	10.1502	down
PARD3	-0.5679	-6.1643	＜0.0001	＜0.0001	10.1278	down
TXNDC17	0.5691	6.1626	＜0.0001	＜0.0001	10.1195	up
GDPD3	0.8151	6.1605	＜0.0001	＜0.0001	10.1094	up
NCAPH	0.8358	6.1539	＜0.0001	＜0.0001	10.0774	up
IRAK2	0.6828	6.1521	＜0.0001	＜0.0001	10.0687	up
MTERF2	-0.6653	-6.1515	＜0.0001	＜0.0001	10.0657	down
MCM10	0.6799	6.1461	＜0.0001	＜0.0001	10.0389	up
ALDH6A1	-0.6317	-6.1444	＜0.0001	＜0.0001	10.0308	down

ETV7	-0.8453	-6.1428	＜0.0001	＜0.0001	10.0231	down
ASF1B	0.7241	6.1422	＜0.0001	＜0.0001	10.0203	up
PIK3C2G	-0.6494	-6.1404	＜0.0001	＜0.0001	10.0114	down
NHLH2	-0.6747	-6.1401	＜0.0001	＜0.0001	10.0098	down
IGFL1	1.4346	6.1389	＜0.0001	＜0.0001	10.0043	up
XPOT	0.5294	6.1379	＜0.0001	＜0.0001	9.9991	up
LINC01805	-0.8358	-6.1297	＜0.0001	＜0.0001	9.9595	down
RCC1	0.7131	6.1297	＜0.0001	＜0.0001	9.9591	up
LINC01550	-0.8036	-6.1293	＜0.0001	＜0.0001	9.9571	down
LYZ	1.2178	6.1225	＜0.0001	＜0.0001	9.9244	up
ZRANB2	-0.5191	-6.1198	＜0.0001	＜0.0001	9.9109	down
ZNF737	-0.6488	-6.1161	＜0.0001	＜0.0001	9.8932	down
PRSS3	0.7671	6.1158	＜0.0001	＜0.0001	9.8918	up
TGM6	0.8969	6.1082	＜0.0001	＜0.0001	9.8546	up
LARGE2	-0.6243	-6.1042	＜0.0001	＜0.0001	9.8354	down
MELK	0.8014	6.0998	＜0.0001	＜0.0001	9.8141	up
DLGAP5	0.8244	6.0984	＜0.0001	＜0.0001	9.8073	up
GDPD2	-0.8357	-6.0953	＜0.0001	＜0.0001	9.7922	down
S100A12	1.5168	6.0865	＜0.0001	＜0.0001	9.7492	up
COBL	-0.5681	-6.0812	＜0.0001	＜0.0001	9.7235	down
HSD17B13	-0.8175	-6.0802	＜0.0001	＜0.0001	9.7188	down
MAP1B	-0.6427	-6.0756	＜0.0001	＜0.0001	9.6964	down
PARP9	0.7811	6.0728	＜0.0001	＜0.0001	9.6833	up
TMEM241	0.7182	6.0704	＜0.0001	＜0.0001	9.6714	up
GALK1	0.5120	6.0645	＜0.0001	＜0.0001	9.6428	up
ACP7	0.9204	6.0628	＜0.0001	＜0.0001	9.6349	up
CXCR1	0.8900	6.0581	＜0.0001	＜0.0001	9.6119	up
MAST1	-0.9144	-6.0538	＜0.0001	＜0.0001	9.5912	down
EPHB1	-0.6452	-6.0522	＜0.0001	＜0.0001	9.5837	down
MAGI1	-0.5217	-6.0522	＜0.0001	＜0.0001	9.5833	down
PNPLA3	-0.8246	-6.0449	＜0.0001	＜0.0001	9.5480	down
DLK2	-0.7504	-6.0394	＜0.0001	＜0.0001	9.5216	down
LAMB4	-0.6727	-6.0387	＜0.0001	＜0.0001	9.5184	down
FRMD8	0.5091	6.0326	＜0.0001	＜0.0001	9.4887	up
KRT6B	2.1905	6.0235	＜0.0001	＜0.0001	9.4448	up
CKMT1A	0.5982	6.0184	＜0.0001	＜0.0001	9.4204	up
GCNT4	0.6989	6.0100	＜0.0001	＜0.0001	9.3802	up
CBR3	0.6467	6.0084	＜0.0001	＜0.0001	9.3723	up
RUFY4	0.8057	6.0040	＜0.0001	＜0.0001	9.3514	up
PGRMC2	-0.5006	-5.9982	＜0.0001	＜0.0001	9.3232	down
RNF222	0.7135	5.9933	＜0.0001	＜0.0001	9.2999	up
SLC4A11	0.5319	5.9918	＜0.0001	＜0.0001	9.2924	up
ANKRD2	-1.0208	-5.9911	＜0.0001	＜0.0001	9.2892	down
TWF1	0.7552	5.9887	＜0.0001	＜0.0001	9.2778	up

CYP4F22	0.5777	5.9863	＜0.0001	＜0.0001	9.2661	up
P2RY2	0.6756	5.9822	＜0.0001	＜0.0001	9.2467	up
MYEOV	-0.7312	-5.9794	＜0.0001	＜0.0001	9.2331	down
ODF3B	0.5989	5.9678	＜0.0001	＜0.0001	9.1776	up
AQP3	0.6350	5.9657	＜0.0001	＜0.0001	9.1676	up
PLSCR1	0.6991	5.9610	＜0.0001	＜0.0001	9.1450	up
FAM189A2	-0.7441	-5.9594	＜0.0001	＜0.0001	9.1373	down
CARHSP1	0.7247	5.9565	＜0.0001	＜0.0001	9.1233	up
DTX3L	0.5913	5.9557	＜0.0001	＜0.0001	9.1195	up
CKS2	0.9390	5.9546	＜0.0001	＜0.0001	9.1143	up
LONRF1	-0.6845	-5.9527	＜0.0001	＜0.0001	9.1052	down
PGF	0.7375	5.9469	＜0.0001	＜0.0001	9.0776	up
FOXQ1	-0.7758	-5.9432	＜0.0001	＜0.0001	9.0599	down
SLC38A5	0.6136	5.9363	＜0.0001	＜0.0001	9.0271	up
LINC00665	-0.5805	-5.9361	＜0.0001	＜0.0001	9.0262	down
PSAPL1	-0.9663	-5.9327	＜0.0001	＜0.0001	9.0095	down
PNISR	-0.5055	-5.9313	＜0.0001	＜0.0001	9.0032	down
POC1A	0.7055	5.9313	＜0.0001	＜0.0001	9.0030	up
OVCH2	-0.7474	-5.9290	＜0.0001	＜0.0001	8.9919	down
CTSD	0.5193	5.9289	＜0.0001	＜0.0001	8.9914	up
GAS1	-0.5619	-5.9281	＜0.0001	＜0.0001	8.9877	down
GALT	-0.5251	-5.9238	＜0.0001	＜0.0001	8.9675	down
NEU2	0.9161	5.9225	＜0.0001	＜0.0001	8.9610	up
CMYA5	-0.7869	-5.9205	＜0.0001	＜0.0001	8.9517	down
ANKRD36B	-0.6357	-5.9198	＜0.0001	＜0.0001	8.9484	down
EPPK1	-0.6889	-5.9149	＜0.0001	＜0.0001	8.9247	down
CPM	-0.6087	-5.9120	＜0.0001	＜0.0001	8.9110	down
NCAPG	0.7221	5.9058	＜0.0001	＜0.0001	8.8813	up
SLC25A25	0.7382	5.9017	＜0.0001	＜0.0001	8.8622	up
DGAT2	-0.7421	-5.8996	＜0.0001	＜0.0001	8.8523	down
PDZD2	-0.5564	-5.8833	＜0.0001	＜0.0001	8.7745	down
MYO7B	-0.7073	-5.8810	＜0.0001	＜0.0001	8.7636	down
OAS2	0.8976	5.8757	＜0.0001	＜0.0001	8.7387	up
HPDL	0.8315	5.8703	＜0.0001	＜0.0001	8.7130	up
TBC1D24	-0.5309	-5.8629	＜0.0001	＜0.0001	8.6779	down
CDH26	0.8617	5.8611	＜0.0001	＜0.0001	8.6692	up
ZYX	0.5313	5.8606	＜0.0001	＜0.0001	8.6670	up
LTF	2.1408	5.8550	＜0.0001	＜0.0001	8.6404	up
AEN	0.7608	5.8421	＜0.0001	＜0.0001	8.5794	up
EFNA5	-0.5282	-5.8337	＜0.0001	＜0.0001	8.5399	down
LGALS9C	0.6063	5.8310	＜0.0001	＜0.0001	8.5270	up
ZNF256	-0.5368	-5.8294	＜0.0001	＜0.0001	8.5195	down
MKI67	0.8051	5.8260	＜0.0001	＜0.0001	8.5035	up
CRYAB	-0.7841	-5.8129	＜0.0001	＜0.0001	8.4418	down

SERPINA1	1.0748	5.8126	＜0.0001	＜0.0001	8.4405	up
DLG2	-0.7274	-5.8126	＜0.0001	＜0.0001	8.4402	down
LAMTOR4	-0.6934	-5.7977	＜0.0001	＜0.0001	8.3701	down
CDH3	0.9565	5.7964	＜0.0001	＜0.0001	8.3641	up
ATAD3A	0.5731	5.7907	＜0.0001	＜0.0001	8.3373	up
HAS3	1.0308	5.7867	＜0.0001	＜0.0001	8.3184	up
CD164L2	0.6660	5.7853	＜0.0001	＜0.0001	8.3120	up
BUB1	0.8779	5.7826	＜0.0001	＜0.0001	8.2994	up
LYPD6B	-0.6000	-5.7795	＜0.0001	＜0.0001	8.2845	down
KCNJ15	0.7052	5.7793	＜0.0001	＜0.0001	8.2839	up
HK2	0.9752	5.7784	＜0.0001	＜0.0001	8.2794	up
EGFR	-0.5491	-5.7727	＜0.0001	＜0.0001	8.2528	down
LMNB2	0.5297	5.7713	＜0.0001	＜0.0001	8.2461	up
TMEM254	-0.5273	-5.7708	＜0.0001	＜0.0001	8.2440	down
CLCA2	0.7064	5.7692	＜0.0001	＜0.0001	8.2366	up
ZNF154	-0.5378	-5.7620	＜0.0001	＜0.0001	8.2026	down
ABCC3	-0.6209	-5.7592	＜0.0001	＜0.0001	8.1897	down
GSTM3	-0.7596	-5.7577	＜0.0001	＜0.0001	8.1826	down
CHRNA9	0.9510	5.7557	＜0.0001	＜0.0001	8.1730	up
DYNLT3	0.5200	5.7537	＜0.0001	＜0.0001	8.1640	up
CLEC10A	0.9748	5.7501	＜0.0001	＜0.0001	8.1470	up
CYB5R4	0.5324	5.7499	＜0.0001	＜0.0001	8.1458	up
PELI2	-0.5043	-5.7429	＜0.0001	＜0.0001	8.1134	down
SYNE2	-0.5031	-5.7388	＜0.0001	＜0.0001	8.0943	down
IL20RB	-0.5179	-5.7305	＜0.0001	＜0.0001	8.0552	down
LAT2	0.5358	5.7294	＜0.0001	＜0.0001	8.0501	up
ZNF91	-0.5327	-5.7294	＜0.0001	＜0.0001	8.0500	down
PALLD	0.5837	5.7248	＜0.0001	＜0.0001	8.0286	up
ELK3	0.7209	5.7167	＜0.0001	＜0.0001	7.9911	up
KLK10	0.7459	5.7163	＜0.0001	＜0.0001	7.9891	up
PTPN21	-0.6939	-5.7137	＜0.0001	＜0.0001	7.9769	down
IFI6	1.0158	5.7104	＜0.0001	＜0.0001	7.9615	up
PACRG	-0.6957	-5.7093	＜0.0001	＜0.0001	7.9566	down
DEPDC1B	0.5788	5.7002	＜0.0001	＜0.0001	7.9142	up
CALML3	0.8925	5.6966	＜0.0001	＜0.0001	7.8974	up
SLC27A4	0.5343	5.6875	＜0.0001	＜0.0001	7.8550	up
TP53INP2	0.6677	5.6859	＜0.0001	＜0.0001	7.8479	up
PRSS22	1.0137	5.6800	＜0.0001	＜0.0001	7.8201	up
DHFR	0.6423	5.6764	＜0.0001	＜0.0001	7.8034	up
FAM117A	-0.5171	-5.6735	＜0.0001	＜0.0001	7.7900	down
LYRM9	-0.5272	-5.6696	＜0.0001	＜0.0001	7.7720	down
MAFTRR	-0.5966	-5.6609	＜0.0001	＜0.0001	7.7315	down
SAMD9	0.7279	5.6564	＜0.0001	＜0.0001	7.7109	up
SKA3	0.7536	5.6515	＜0.0001	＜0.0001	7.6884	up

ZNF471	-0.5061	-5.6514	＜0.0001	＜0.0001	7.6876	down
H1-1	0.5164	5.6435	＜0.0001	＜0.0001	7.6514	up
CKMT1B	0.5667	5.6392	＜0.0001	＜0.0001	7.6311	up
ZC3H12A	0.7932	5.6388	＜0.0001	＜0.0001	7.6296	up
HS3ST3A1	0.7133	5.6273	＜0.0001	＜0.0001	7.5765	up
PBK	0.8714	5.6267	＜0.0001	＜0.0001	7.5738	up
SYT8	-1.1357	-5.6256	＜0.0001	＜0.0001	7.5685	down
MIR3936HG	-0.5429	-5.6234	＜0.0001	＜0.0001	7.5585	down
PIF1	0.7528	5.6233	＜0.0001	＜0.0001	7.5580	up
FHL1	-0.9043	-5.6225	＜0.0001	＜0.0001	7.5545	down
ASPA	-0.5948	-5.6070	＜0.0001	＜0.0001	7.4831	down
CDC45	0.7918	5.5994	＜0.0001	＜0.0001	7.4480	up
MSMB	-1.2847	-5.5907	＜0.0001	＜0.0001	7.4077	down
LCE2B	-0.8064	-5.5887	＜0.0001	＜0.0001	7.3986	down
COL6A6	1.2370	5.5868	＜0.0001	＜0.0001	7.3899	up
PBX3	-0.5062	-5.5838	＜0.0001	＜0.0001	7.3761	down
IL22RA1	-0.5072	-5.5821	＜0.0001	＜0.0001	7.3683	down
BZW1	0.5495	5.5781	＜0.0001	＜0.0001	7.3499	up
CENPN	0.5065	5.5780	＜0.0001	＜0.0001	7.3496	up
VWF	0.6877	5.5772	＜0.0001	＜0.0001	7.3458	up
LINC01133	-0.6079	-5.5766	＜0.0001	＜0.0001	7.3434	down
SLC8A1	-0.8196	-5.5727	＜0.0001	＜0.0001	7.3256	down
PON3	-0.7464	-5.5654	＜0.0001	＜0.0001	7.2921	down
CLSPN	0.5120	5.5618	＜0.0001	＜0.0001	7.2755	up
FBLIM1	0.7731	5.5588	＜0.0001	＜0.0001	7.2615	up
WDR4	0.6220	5.5576	＜0.0001	＜0.0001	7.2563	up
TRIM15	0.8045	5.5546	＜0.0001	＜0.0001	7.2424	up
CDHR1	-0.7001	-5.5521	＜0.0001	＜0.0001	7.2311	down
KLK13	0.9635	5.5483	＜0.0001	＜0.0001	7.2136	up
RHCG	1.5878	5.5449	＜0.0001	＜0.0001	7.1981	up
BHLHE40	0.9431	5.5447	＜0.0001	＜0.0001	7.1972	up
EDA	-0.5418	-5.5362	＜0.0001	＜0.0001	7.1583	down
CTPS1	0.6335	5.5353	＜0.0001	＜0.0001	7.1544	up
TNNC1	-0.6625	-5.5342	＜0.0001	＜0.0001	7.1494	down
POU6F1	-0.5220	-5.5282	＜0.0001	＜0.0001	7.1217	down
SLC35C1	0.5314	5.5259	＜0.0001	＜0.0001	7.1115	up
ERCC6L	0.5158	5.5160	＜0.0001	＜0.0001	7.0665	up
FAM221A	-0.5244	-5.4988	＜0.0001	＜0.0001	6.9880	down
LINC00885	-0.7210	-5.4946	＜0.0001	＜0.0001	6.9691	down
MEGF6	-0.5642	-5.4786	＜0.0001	＜0.0001	6.8965	down
SNCG	-0.6844	-5.4777	＜0.0001	＜0.0001	6.8923	down
RTP4	0.6574	5.4773	＜0.0001	＜0.0001	6.8906	up
LIPA	0.5821	5.4766	＜0.0001	＜0.0001	6.8874	up
RAB40C	-0.5735	-5.4717	＜0.0001	＜0.0001	6.8650	down

WNT5A	0.6577	5.4691	＜0.0001	＜0.0001	6.8535	up
SNX10	0.6525	5.4627	＜0.0001	＜0.0001	6.8246	up
SYT12	0.6301	5.4582	＜0.0001	＜0.0001	6.8040	up
LINC01229	-0.5349	-5.4562	＜0.0001	＜0.0001	6.7952	down
KIF4A	0.9659	5.4547	＜0.0001	＜0.0001	6.7883	up
SLC20A1	0.6139	5.4502	＜0.0001	＜0.0001	6.7680	up
CA3	-0.6542	-5.4493	＜0.0001	＜0.0001	6.7639	down
H2AX	0.5220	5.4447	＜0.0001	＜0.0001	6.7429	up
CHRM1	-0.5901	-5.4437	＜0.0001	＜0.0001	6.7388	down
PSORS1C2	-0.8994	-5.4437	＜0.0001	＜0.0001	6.7384	down
SYBU	-0.5893	-5.4423	＜0.0001	＜0.0001	6.7324	down
IFI44	0.9085	5.4371	＜0.0001	＜0.0001	6.7087	up
GPR22	-0.5172	-5.4354	＜0.0001	＜0.0001	6.7013	down
GOLGA8B	-0.5155	-5.4348	＜0.0001	＜0.0001	6.6983	down
TACSTD2	-0.5192	-5.4337	＜0.0001	＜0.0001	6.6936	down
FBXL16	-0.6493	-5.4326	＜0.0001	＜0.0001	6.6887	down
SCRG1	-0.5355	-5.4276	＜0.0001	＜0.0001	6.6659	down
RHCE	0.5754	5.4274	＜0.0001	＜0.0001	6.6653	up
EPGN	1.1106	5.4237	＜0.0001	＜0.0001	6.6482	up
STIL	0.6222	5.4170	＜0.0001	＜0.0001	6.6183	up
LINC01300	-0.6064	-5.4167	＜0.0001	＜0.0001	6.6171	down
BEST4	-0.6932	-5.4151	＜0.0001	＜0.0001	6.6097	down
UHRF1	0.7460	5.4104	＜0.0001	＜0.0001	6.5884	up
AAMDC	-0.6379	-5.4088	＜0.0001	＜0.0001	6.5813	down
RELT	0.5369	5.4087	＜0.0001	＜0.0001	6.5808	up
MAD2L1	0.5230	5.4085	＜0.0001	＜0.0001	6.5801	up
AMD1	0.5463	5.4077	＜0.0001	＜0.0001	6.5764	up
R3HDM4	0.5681	5.4050	＜0.0001	＜0.0001	6.5643	up
EPHA2	0.5606	5.4023	＜0.0001	＜0.0001	6.5525	up
TSC22D3	-0.7654	-5.4013	＜0.0001	＜0.0001	6.5479	down
KRT31	-1.3133	-5.3984	＜0.0001	＜0.0001	6.5348	down
F2R	-0.7364	-5.3976	＜0.0001	＜0.0001	6.5310	down
FCGBP	-0.9794	-5.3931	＜0.0001	＜0.0001	6.5109	down
PLEKHG2	0.5261	5.3929	＜0.0001	＜0.0001	6.5101	up
FAM83A	0.8632	5.3909	＜0.0001	＜0.0001	6.5012	up
PACC1	0.5027	5.3873	＜0.0001	＜0.0001	6.4848	up
KLK1	-0.8205	-5.3832	＜0.0001	＜0.0001	6.4668	down
SUGCT	0.5094	5.3813	＜0.0001	＜0.0001	6.4580	up
CRABP2	0.5790	5.3798	＜0.0001	＜0.0001	6.4515	up
NRBP2	-0.5417	-5.3736	＜0.0001	＜0.0001	6.4237	down
SLC6A14	0.9643	5.3673	＜0.0001	＜0.0001	6.3955	up
ZNF391	-0.6152	-5.3672	＜0.0001	＜0.0001	6.3951	down
TPX2	0.5844	5.3606	＜0.0001	＜0.0001	6.3656	up
RFTN1	0.6540	5.3605	＜0.0001	＜0.0001	6.3651	up

GPX3	1.1057	5.3600	＜0.0001	＜0.0001	6.3628	up
WAKMAR2	-0.6993	-5.3489	＜0.0001	＜0.0001	6.3131	down
GPX2	0.7672	5.3467	＜0.0001	＜0.0001	6.3033	up
AHNAK2	-0.5133	-5.3459	＜0.0001	＜0.0001	6.2998	down
MIR4435-2HG	0.5344	5.3442	＜0.0001	＜0.0001	6.2922	up
GNA15	0.5822	5.3436	＜0.0001	＜0.0001	6.2899	up
CSTA	0.5714	5.3426	＜0.0001	＜0.0001	6.2854	up
TBXAS1	0.7882	5.3317	＜0.0001	＜0.0001	6.2366	up
TACC3	0.7363	5.3211	＜0.0001	＜0.0001	6.1894	up
GALNT18	0.6103	5.3134	＜0.0001	＜0.0001	6.1556	up
ACSF2	-0.5351	-5.3092	＜0.0001	＜0.0001	6.1366	down
KIF23	0.7050	5.3043	＜0.0001	＜0.0001	6.1148	up
MT1X	-0.7332	-5.3015	＜0.0001	＜0.0001	6.1028	down
CHRNE	-0.7301	-5.2965	＜0.0001	＜0.0001	6.0802	down
C12orf56	0.5533	5.2957	＜0.0001	＜0.0001	6.0768	up
ILDR1	-0.5352	-5.2952	＜0.0001	＜0.0001	6.0745	down
KIF20A	0.7888	5.2916	＜0.0001	＜0.0001	6.0586	up
RNF227	-0.5402	-5.2737	＜0.0001	＜0.0001	5.9795	down
CYSRT1	0.5380	5.2724	＜0.0001	＜0.0001	5.9739	up
DAAM1	-0.5032	-5.2678	＜0.0001	＜0.0001	5.9536	down
ADAM8	0.6501	5.2678	＜0.0001	＜0.0001	5.9535	up
COL4A1	0.8293	5.2673	＜0.0001	＜0.0001	5.9514	up
TCIRG1	0.5474	5.2666	＜0.0001	＜0.0001	5.9484	up
USP43	0.5450	5.2632	＜0.0001	＜0.0001	5.9332	up
PLEKHG5	-0.5303	-5.2561	＜0.0001	＜0.0001	5.9020	down
AREG	0.8678	5.2506	＜0.0001	＜0.0001	5.8779	up
PDZK1IP1	0.5214	5.2475	＜0.0001	＜0.0001	5.8642	up
RARRES1	-0.9884	-5.2278	＜0.0001	＜0.0001	5.7777	down
CXCR6	1.0318	5.2277	＜0.0001	＜0.0001	5.7772	up
SASH3	0.8073	5.2261	＜0.0001	＜0.0001	5.7702	up
ADAM19	1.0199	5.2234	＜0.0001	＜0.0001	5.7584	up
IL4I1	1.0345	5.2227	＜0.0001	＜0.0001	5.7552	up
MTFR2	0.5617	5.2222	＜0.0001	＜0.0001	5.7529	up
SOCS3	1.0323	5.2187	＜0.0001	＜0.0001	5.7376	up
HAPLN3	0.6171	5.2186	＜0.0001	＜0.0001	5.7373	up
ELMOD1	-0.6480	-5.2162	＜0.0001	＜0.0001	5.7264	down
SH3TC1	0.5951	5.2117	＜0.0001	＜0.0001	5.7070	up
KY	-0.5122	-5.2109	＜0.0001	＜0.0001	5.7034	down
LCE3C	2.0997	5.2047	＜0.0001	＜0.0001	5.6761	up
PCSK9	0.5745	5.1996	＜0.0001	＜0.0001	5.6540	up
PLBD1	0.6127	5.1994	＜0.0001	＜0.0001	5.6530	up
ZNF273	-0.6802	-5.1989	＜0.0001	＜0.0001	5.6506	down
TNNI2	-0.9605	-5.1969	＜0.0001	＜0.0001	5.6423	down
CYP27B1	0.6552	5.1879	＜0.0001	＜0.0001	5.6030	up

ADAM23	0.6315	5.1876	＜0.0001	＜0.0001	5.6014	up
PNPLA7	-0.5948	-5.1855	＜0.0001	＜0.0001	5.5923	down
APOL6	0.6498	5.1853	＜0.0001	＜0.0001	5.5916	up
HLA-DQB2	-0.7455	-5.1848	＜0.0001	＜0.0001	5.5893	down
ETS1	0.5670	5.1835	＜0.0001	＜0.0001	5.5835	up
NRXN3	-0.5330	-5.1794	＜0.0001	＜0.0001	5.5658	down
RIMS3	0.8124	5.1781	＜0.0001	＜0.0001	5.5600	up
CTSV	-0.7988	-5.1734	＜0.0001	＜0.0001	5.5396	down
FAIM2	0.9209	5.1669	＜0.0001	＜0.0001	5.5110	up
FLG	-0.8893	-5.1661	＜0.0001	＜0.0001	5.5077	down
ANKRD29	0.7193	5.1633	＜0.0001	＜0.0001	5.4957	up
SORBS1	-0.6412	-5.1566	＜0.0001	＜0.0001	5.4663	down
ADAMTSL3	-0.6943	-5.1549	＜0.0001	＜0.0001	5.4592	down
SRCIN1	-0.5510	-5.1532	＜0.0001	＜0.0001	5.4515	down
EPSTI1	0.8667	5.1510	＜0.0001	＜0.0001	5.4422	up
CTSH	-0.6022	-5.1459	＜0.0001	＜0.0001	5.4200	down
PDZD7	-0.6012	-5.1457	＜0.0001	＜0.0001	5.4191	down
PLA2G3	0.7570	5.1451	＜0.0001	＜0.0001	5.4164	up
CCR7	0.9310	5.1442	＜0.0001	＜0.0001	5.4125	up
KIF11	0.5754	5.1418	＜0.0001	＜0.0001	5.4020	up
PIK3R1	-0.5245	-5.1415	＜0.0001	＜0.0001	5.4007	down
AGFG2	-0.5899	-5.1412	＜0.0001	＜0.0001	5.3996	down
COMP	1.1553	5.1404	＜0.0001	＜0.0001	5.3960	up
CDC20	0.8088	5.1364	＜0.0001	＜0.0001	5.3788	up
MYH14	-0.6537	-5.1357	＜0.0001	＜0.0001	5.3755	down
SLC26A9	0.9246	5.1337	＜0.0001	＜0.0001	5.3672	up
NDC80	0.5709	5.1319	＜0.0001	＜0.0001	5.3593	up
PUDP	0.5275	5.1239	＜0.0001	＜0.0001	5.3244	up
GPNMB	-0.5230	-5.1234	＜0.0001	＜0.0001	5.3224	down
UPP1	1.2223	5.1213	＜0.0001	＜0.0001	5.3132	up
CDK1	0.6920	5.1195	＜0.0001	＜0.0001	5.3054	up
GAL3ST4	-0.5235	-5.1174	＜0.0001	＜0.0001	5.2965	down
IL17RE	-0.5154	-5.1171	＜0.0001	＜0.0001	5.2951	down
KIF2C	0.6519	5.1148	＜0.0001	＜0.0001	5.2853	up
CLIC4	0.5744	5.1131	＜0.0001	＜0.0001	5.2777	up
CDKN3	0.9795	5.1124	＜0.0001	＜0.0001	5.2749	up
TBILA	-0.5702	-5.1090	＜0.0001	＜0.0001	5.2599	down
LCE1A	-0.7825	-5.1076	＜0.0001	＜0.0001	5.2539	down
BTAF1	-0.5984	-5.1069	＜0.0001	＜0.0001	5.2510	down
KCTD11	0.6794	5.1061	＜0.0001	＜0.0001	5.2478	up
CD1B	1.4273	5.1013	＜0.0001	＜0.0001	5.2271	up
LDLR	0.6963	5.0969	＜0.0001	＜0.0001	5.2078	up
C1QTNF1	0.5121	5.0907	＜0.0001	＜0.0001	5.1813	up
TIMM10	0.5015	5.0859	＜0.0001	＜0.0001	5.1605	up

CD28	0.7903	5.0854	＜0.0001	＜0.0001	5.1583	up
AURKA	0.6051	5.0805	＜0.0001	＜0.0001	5.1371	up
TNIP3	0.8379	5.0783	＜0.0001	＜0.0001	5.1276	up
ADAM9	0.5938	5.0766	＜0.0001	＜0.0001	5.1204	up
OR2A7	-0.5871	-5.0755	＜0.0001	＜0.0001	5.1156	down
USP38	0.5486	5.0743	＜0.0001	＜0.0001	5.1106	up
RSAD2	0.9455	5.0739	＜0.0001	＜0.0001	5.1090	up
P2RY1	0.5290	5.0651	＜0.0001	＜0.0001	5.0711	up
IL20RA	-0.5962	-5.0650	＜0.0001	＜0.0001	5.0705	down
LYNX1-SLURP2	-0.8689	-5.0630	＜0.0001	＜0.0001	5.0621	down
TROAP	0.5454	5.0607	＜0.0001	＜0.0001	5.0523	up
GTSE1	0.5965	5.0605	＜0.0001	＜0.0001	5.0514	up
ANKFN1	-0.7067	-5.0550	＜0.0001	＜0.0001	5.0279	down
TMPRSS11D	1.0093	5.0526	＜0.0001	＜0.0001	5.0176	up
GSPT2	-0.5420	-5.0521	＜0.0001	＜0.0001	5.0153	down
UBE2S	0.5078	5.0496	＜0.0001	＜0.0001	5.0047	up
CLDN1	-0.6246	-5.0492	＜0.0001	＜0.0001	5.0027	down
KIAA1217	0.5005	5.0486	＜0.0001	＜0.0001	5.0005	up
SYNPO2	-0.7452	-5.0485	＜0.0001	＜0.0001	5.0000	down
LRP8	0.9092	5.0439	＜0.0001	＜0.0001	4.9804	up
CDC6	0.6045	5.0439	＜0.0001	＜0.0001	4.9803	up
SKA1	0.6505	5.0428	＜0.0001	＜0.0001	4.9756	up
ATP12A	1.3943	5.0378	＜0.0001	＜0.0001	4.9542	up
NPM3	0.5563	5.0319	＜0.0001	＜0.0001	4.9290	up
CTSL	0.6847	5.0300	＜0.0001	＜0.0001	4.9206	up
CA13	-0.5914	-5.0262	＜0.0001	＜0.0001	4.9046	down
UST	-0.5444	-5.0259	＜0.0001	＜0.0001	4.9034	down
SP8	-0.6614	-5.0212	＜0.0001	＜0.0001	4.8830	down
CA2	0.7762	5.0203	＜0.0001	＜0.0001	4.8795	up
KIF18A	0.6668	5.0201	＜0.0001	＜0.0001	4.8787	up
MROH2A	-0.5780	-5.0182	＜0.0001	＜0.0001	4.8703	down
SPTLC2	0.6589	5.0145	＜0.0001	＜0.0001	4.8545	up
EPHB2	0.6029	5.0133	＜0.0001	＜0.0001	4.8494	up
MINCR	-0.5793	-5.0109	＜0.0001	＜0.0001	4.8392	down
LINC02159	0.5643	5.0102	＜0.0001	＜0.0001	4.8361	up
ECT2	0.7022	5.0090	＜0.0001	＜0.0001	4.8313	up
FCMR	0.5400	5.0074	＜0.0001	＜0.0001	4.8245	up
TMEM271	-0.6400	-5.0052	＜0.0001	＜0.0001	4.8149	down
KLK6	1.7649	4.9935	＜0.0001	＜0.0001	4.7652	up
AURKB	0.6221	4.9917	＜0.0001	＜0.0001	4.7576	up
SLC18A2	-0.5063	-4.9880	＜0.0001	＜0.0001	4.7420	down
PPP1R14C	-0.5075	-4.9807	＜0.0001	＜0.0001	4.7108	down
HYAL4	0.5348	4.9766	＜0.0001	＜0.0001	4.6935	up
GAS6	-0.5738	-4.9763	＜0.0001	＜0.0001	4.6924	down

CCNA2	0.7770	4.9733	＜0.0001	＜0.0001	4.6796	up
PKIB	-0.6844	-4.9671	＜0.0001	＜0.0001	4.6533	down
SRGN	0.7954	4.9640	＜0.0001	＜0.0001	4.6400	up
ZC3H12C	0.5971	4.9583	＜0.0001	＜0.0001	4.6159	up
FAM218A	-0.5076	-4.9538	＜0.0001	＜0.0001	4.5972	down
CDCA5	0.5493	4.9502	＜0.0001	＜0.0001	4.5817	up
CDCA2	0.6198	4.9485	＜0.0001	＜0.0001	4.5746	up
ZBED2	0.7974	4.9451	＜0.0001	＜0.0001	4.5601	up
ZWINT	0.5896	4.9423	＜0.0001	＜0.0001	4.5486	up
IL2RA	0.6354	4.9411	＜0.0001	＜0.0001	4.5433	up
ANGPTL2	0.5621	4.9388	＜0.0001	＜0.0001	4.5337	up
MATN2	-0.5010	-4.9337	＜0.0001	＜0.0001	4.5122	down
GBP6	0.9441	4.9320	＜0.0001	＜0.0001	4.5052	up
USP18	0.5573	4.9245	＜0.0001	＜0.0001	4.4734	up
LINC00681	-0.5953	-4.9190	＜0.0001	＜0.0001	4.4502	down
NEK2	0.5762	4.9143	＜0.0001	＜0.0001	4.4307	up
TOP2A	0.7389	4.9091	＜0.0001	＜0.0001	4.4086	up
HUNK	-0.5921	-4.9036	＜0.0001	＜0.0001	4.3855	down
MSN	0.5515	4.8972	＜0.0001	＜0.0001	4.3589	up
STEAP4	1.0713	4.8960	＜0.0001	＜0.0001	4.3536	up
POF1B	-0.5199	-4.8927	＜0.0001	＜0.0001	4.3398	down
PTAFR	0.5308	4.8911	＜0.0001	＜0.0001	4.3334	up
APOL1	0.6724	4.8884	＜0.0001	＜0.0001	4.3221	up
PRR9	2.1251	4.8843	＜0.0001	＜0.0001	4.3050	up
GPR153	0.5128	4.8843	＜0.0001	＜0.0001	4.3049	up
SDC4	-0.5421	-4.8792	＜0.0001	＜0.0001	4.2834	down
PLEKHH1	-0.5386	-4.8725	＜0.0001	＜0.0001	4.2554	down
COTL1	0.6999	4.8713	＜0.0001	＜0.0001	4.2505	up
PAQR5	0.5736	4.8694	＜0.0001	＜0.0001	4.2426	up
TNFRSF12A	0.7954	4.8634	＜0.0001	＜0.0001	4.2177	up
CLDN3	-0.7675	-4.8571	＜0.0001	＜0.0001	4.1912	down
GZMB	1.0064	4.8527	＜0.0001	＜0.0001	4.1728	up
IL11RA	-0.5066	-4.8518	＜0.0001	＜0.0001	4.1694	down
LYN	0.6986	4.8501	＜0.0001	＜0.0001	4.1623	up
CCL22	1.0945	4.8470	＜0.0001	＜0.0001	4.1491	up
HRH2	0.7012	4.8460	＜0.0001	＜0.0001	4.1451	up
IL18	-0.5139	-4.8422	＜0.0001	＜0.0001	4.1291	down
NPY1R	-0.9058	-4.8338	＜0.0001	＜0.0001	4.0946	down
HERC6	0.5999	4.8335	＜0.0001	＜0.0001	4.0933	up
ORC1	0.5879	4.8295	＜0.0001	＜0.0001	4.0764	up
B3GNT5	0.5155	4.8243	＜0.0001	＜0.0001	4.0549	up
PARP14	0.5178	4.8179	＜0.0001	＜0.0001	4.0285	up
NDRG4	0.7430	4.8171	＜0.0001	＜0.0001	4.0252	up
ITM2A	-0.5508	-4.8011	＜0.0001	＜0.0001	3.9588	down

FXYD5	0.6181	4.7877	＜0.0001	＜0.0001	3.9039	up
COL7A1	-0.5430	-4.7863	＜0.0001	＜0.0001	3.8978	down
HBEGF	1.0210	4.7823	＜0.0001	＜0.0001	3.8815	up
LMNB1	0.5952	4.7756	＜0.0001	＜0.0001	3.8540	up
TNFRSF9	0.6845	4.7734	＜0.0001	＜0.0001	3.8447	up
LURAP1L	0.7215	4.7729	＜0.0001	＜0.0001	3.8429	up
ISG15	0.8863	4.7716	＜0.0001	＜0.0001	3.8373	up
PRLR	-0.6736	-4.7676	＜0.0001	＜0.0001	3.8212	down
CYP4F12	-0.5597	-4.7658	＜0.0001	＜0.0001	3.8136	down
SPTBN5	0.7435	4.7543	＜0.0001	＜0.0001	3.7665	up
SIRPG	0.6126	4.7456	＜0.0001	＜0.0001	3.7306	up
CALCRL	0.6816	4.7424	＜0.0001	＜0.0001	3.7177	up
TEX101	0.7027	4.7422	＜0.0001	＜0.0001	3.7167	up
RGS1	0.9174	4.7416	＜0.0001	＜0.0001	3.7146	up
MASP1	0.6164	4.7399	＜0.0001	＜0.0001	3.7075	up
IL1R2	-0.5848	-4.7398	＜0.0001	＜0.0001	3.7071	down
DOK7	-0.6887	-4.7310	＜0.0001	0.0001	3.6710	down
AKR1C3	-0.5428	-4.7284	＜0.0001	0.0001	3.6605	down
CTSF	-0.6250	-4.7254	＜0.0001	0.0001	3.6481	down
CCR1	0.8482	4.7075	＜0.0001	0.0001	3.5752	up
TTK	0.5580	4.7063	＜0.0001	0.0001	3.5704	up
CDC25C	0.5336	4.7026	＜0.0001	0.0001	3.5551	up
IFITM3	0.5417	4.7006	＜0.0001	0.0001	3.5470	up
GSTA3	-0.7693	-4.6967	＜0.0001	0.0001	3.5315	down
CDCA8	0.5916	4.6944	＜0.0001	0.0001	3.5218	up
SERPINB7	-0.5073	-4.6891	＜0.0001	0.0001	3.5003	down
ENKUR	0.6893	4.6825	＜0.0001	0.0001	3.4738	up
SLC47A1	-0.5706	-4.6792	＜0.0001	0.0001	3.4602	down
ALOX15	0.8776	4.6782	＜0.0001	0.0001	3.4563	up
CSF2RB	0.5851	4.6757	＜0.0001	0.0001	3.4459	up
ADCY2	-0.5222	-4.6589	＜0.0001	0.0001	3.3783	down
SAMSN1	0.7238	4.6568	＜0.0001	0.0001	3.3697	up
FPR3	0.5042	4.6481	＜0.0001	0.0001	3.3345	up
CD96	0.6271	4.6462	＜0.0001	0.0001	3.3270	up
PAPLN	-0.5896	-4.6329	＜0.0001	0.0001	3.2733	down
EPHB6	-0.5560	-4.6311	＜0.0001	0.0001	3.2661	down
AZGP1	-0.5376	-4.6273	＜0.0001	0.0001	3.2509	down
NABP1	0.6183	4.6152	＜0.0001	0.0001	3.2023	up
CST7	0.8693	4.6107	＜0.0001	0.0001	3.1842	up
FERMT3	0.5791	4.6055	＜0.0001	0.0001	3.1632	up
MYO1B	0.7368	4.6014	＜0.0001	0.0001	3.1469	up
NUF2	0.6198	4.5943	＜0.0001	0.0001	3.1186	up
TMEM116	-0.5743	-4.5852	＜0.0001	0.0001	3.0822	down
CCNB2	0.8046	4.5776	＜0.0001	0.0001	3.0520	up

CENPW	0.5174	4.5748	＜0.0001	0.0001	3.0409	up
ADAMTS4	0.8126	4.5741	＜0.0001	0.0001	3.0381	up
CYP24A1	0.8567	4.5735	＜0.0001	0.0001	3.0355	up
HEATR4	-0.5259	-4.5733	＜0.0001	0.0001	3.0349	down
SLAMF1	0.5646	4.5728	＜0.0001	0.0001	3.0329	up
CCL13	1.2136	4.5725	＜0.0001	0.0001	3.0317	up
PDK4	-0.6899	-4.5644	＜0.0001	0.0001	2.9992	down
POU3F1	-0.5114	-4.5638	＜0.0001	0.0001	2.9968	down
CYP2S1	0.5973	4.5622	＜0.0001	0.0001	2.9906	up
IFITM1	0.5919	4.5595	＜0.0001	0.0001	2.9799	up
BTBD16	-0.5761	-4.5579	＜0.0001	0.0001	2.9734	down
CD1D	0.6733	4.5570	＜0.0001	0.0001	2.9699	up
KYNU	0.6802	4.5532	＜0.0001	0.0001	2.9548	up
CAMSAP3	-0.5226	-4.5458	＜0.0001	0.0001	2.9254	down
THY1	0.9989	4.5441	＜0.0001	0.0001	2.9190	up
CST6	-0.8726	-4.5430	＜0.0001	0.0001	2.9143	down
CENPF	0.6006	4.5412	＜0.0001	0.0001	2.9073	up
CSRP2	0.7102	4.5353	＜0.0001	0.0001	2.8840	up
MYCL	-0.5421	-4.5266	＜0.0001	0.0001	2.8496	down
SCNN1D	0.6598	4.5204	＜0.0001	0.0001	2.8252	up
TNFRSF4	0.5217	4.5196	＜0.0001	0.0001	2.8219	up
FIBIN	-0.6065	-4.5191	＜0.0001	0.0001	2.8199	down
ASPG	0.5298	4.5158	＜0.0001	0.0001	2.8071	up
TMEM132B	-0.6926	-4.5156	＜0.0001	0.0001	2.8063	down
CRY2	-0.6003	-4.5066	＜0.0001	0.0001	2.7705	down
LIN7B	-0.5082	-4.5061	＜0.0001	0.0001	2.7687	down
GGCT	0.5033	4.4952	＜0.0001	0.0001	2.7259	up
CD5	0.6257	4.4886	＜0.0001	0.0001	2.6997	up
IL7R	0.7320	4.4846	＜0.0001	0.0001	2.6842	up
ASPM	0.5971	4.4801	＜0.0001	0.0001	2.6667	up
GLIS1	-0.5316	-4.4693	＜0.0001	0.0001	2.6240	down
ADH7	0.5804	4.4667	＜0.0001	0.0001	2.6141	up
NPAS1	-0.7172	-4.4580	＜0.0001	0.0001	2.5801	down
JAML	0.6071	4.4505	＜0.0001	0.0001	2.5507	up
LILRA6	1.1640	4.4450	＜0.0001	0.0001	2.5293	up
DOC2B	0.5527	4.4429	＜0.0001	0.0001	2.5209	up
CD53	0.6763	4.4410	＜0.0001	0.0001	2.5135	up
SULF2	0.5752	4.4268	＜0.0001	0.0002	2.4586	up
POU2F3	-0.5185	-4.4251	＜0.0001	0.0002	2.4517	down
APOBEC3A	1.1008	4.4216	＜0.0001	0.0002	2.4383	up
ECRG4	-0.6102	-4.4175	＜0.0001	0.0002	2.4223	down
SELPLG	0.5495	4.4155	＜0.0001	0.0002	2.4145	up
PLAU	0.5572	4.4128	＜0.0001	0.0002	2.4040	up
PYCR1	0.5321	4.4114	＜0.0001	0.0002	2.3986	up

AR	-0.6839	-4.4102	＜0.0001	0.0002	2.3941	down
ZDHHC11B	-0.7229	-4.4101	＜0.0001	0.0002	2.3938	down
PARP12	0.5113	4.4056	＜0.0001	0.0002	2.3763	up
TIMELESS	0.6458	4.4047	＜0.0001	0.0002	2.3727	up
ETHE1	0.5043	4.4022	＜0.0001	0.0002	2.3631	up
GATM	-0.5705	-4.3971	＜0.0001	0.0002	2.3436	down
RELB	0.5488	4.3930	＜0.0001	0.0002	2.3274	up
KRT10	-0.7150	-4.3926	＜0.0001	0.0002	2.3259	down
FCGR3A	0.7327	4.3922	＜0.0001	0.0002	2.3243	up
SPOCK2	0.5598	4.3871	＜0.0001	0.0002	2.3046	up
TNFSF8	0.6365	4.3831	＜0.0001	0.0002	2.2895	up
PLCH2	-0.5595	-4.3799	＜0.0001	0.0002	2.2771	down
RAC2	0.6329	4.3790	＜0.0001	0.0002	2.2734	up
SLC25A29	-0.5068	-4.3753	＜0.0001	0.0002	2.2594	down
COL6A3	0.7070	4.3746	＜0.0001	0.0002	2.2567	up
SERPINB9	0.5868	4.3736	＜0.0001	0.0002	2.2529	up
TNFRSF10B	0.5330	4.3725	＜0.0001	0.0002	2.2484	up
CD207	-0.7461	-4.3716	＜0.0001	0.0002	2.2451	down
CD3G	0.6534	4.3682	＜0.0001	0.0002	2.2318	up
VILL	-0.5048	-4.3612	＜0.0001	0.0002	2.2049	down
TMEM86A	0.5071	4.3609	＜0.0001	0.0002	2.2038	up
CD1A	-0.6712	-4.3569	＜0.0001	0.0002	2.1885	down
LGALS3BP	0.5208	4.3522	＜0.0001	0.0002	2.1704	up
FABP7	-1.2605	-4.3485	＜0.0001	0.0002	2.1565	down
DAW1	-0.7257	-4.3484	＜0.0001	0.0002	2.1561	down
NRP2	0.5492	4.3406	＜0.0001	0.0002	2.1261	up
TPM1	-0.5727	-4.3399	＜0.0001	0.0002	2.1233	down
DDIAS	0.5431	4.3342	＜0.0001	0.0002	2.1017	up
LGALS2	0.7068	4.3304	＜0.0001	0.0002	2.0872	up
CADM2	-0.5124	-4.3277	＜0.0001	0.0002	2.0770	down
SEC14L6	-1.5267	-4.3266	＜0.0001	0.0002	2.0725	down
CES1	-0.7345	-4.3240	＜0.0001	0.0002	2.0625	down
LINC02175	-0.6712	-4.3136	＜0.0001	0.0002	2.0229	down
RND3	0.6050	4.3037	＜0.0001	0.0002	1.9855	up
PLA2G2A	1.2761	4.3025	＜0.0001	0.0002	1.9810	up
ADRB2	-0.6163	-4.3017	＜0.0001	0.0002	1.9779	down
FBLN1	-0.7585	-4.2994	＜0.0001	0.0002	1.9691	down
VEGFA	0.5460	4.2977	＜0.0001	0.0002	1.9626	up
PSCA	-0.6654	-4.2921	＜0.0001	0.0002	1.9414	down
APOD	-0.8364	-4.2877	＜0.0001	0.0002	1.9247	down
OR10A2	0.7048	4.2848	＜0.0001	0.0002	1.9135	up
IL32	0.7560	4.2813	＜0.0001	0.0002	1.9004	up
RASGRP1	0.5891	4.2808	＜0.0001	0.0003	1.8985	up
TMC6	0.5002	4.2805	＜0.0001	0.0003	1.8973	up

CCL20	1.1290	4.2768	＜0.0001	0.0003	1.8834	up
TNNT3	-0.6024	-4.2698	＜0.0001	0.0003	1.8569	down
KRT17	1.3889	4.2652	＜0.0001	0.0003	1.8395	up
HRNR	1.1865	4.2632	＜0.0001	0.0003	1.8322	up
IGFL2	-0.7880	-4.2514	＜0.0001	0.0003	1.7876	down
NCAPD2	0.5241	4.2440	＜0.0001	0.0003	1.7598	up
GNLY	0.6814	4.2422	＜0.0001	0.0003	1.7529	up
ANPEP	0.5495	4.2419	＜0.0001	0.0003	1.7519	up
UBE2C	0.6871	4.2412	＜0.0001	0.0003	1.7491	up
FAM111B	0.5775	4.2333	＜0.0001	0.0003	1.7195	up
ZNF165	0.5694	4.2332	＜0.0001	0.0003	1.7191	up
LCK	0.7949	4.2138	＜0.0001	0.0003	1.6467	up
CYP4B1	-0.8425	-4.2046	＜0.0001	0.0003	1.6120	down
ARSF	0.6274	4.2029	＜0.0001	0.0003	1.6059	up
IFIT3	0.6704	4.1953	＜0.0001	0.0003	1.5776	up
EPS8L1	-0.5091	-4.1887	＜0.0001	0.0003	1.5530	down
IRF8	0.6654	4.1851	＜0.0001	0.0003	1.5395	up
TLCD1	-0.5215	-4.1741	＜0.0001	0.0004	1.4987	down
TRBC2	0.7428	4.1704	0.0001	0.0004	1.4850	up
CYTH4	0.6649	4.1702	0.0001	0.0004	1.4841	up
CA9	-0.5568	-4.1691	0.0001	0.0004	1.4800	down
KIFC1	0.6751	4.1671	0.0001	0.0004	1.4729	up
PTP4A3	0.5278	4.1536	0.0001	0.0004	1.4226	up
BGN	-0.5886	-4.1496	0.0001	0.0004	1.4078	down
SELL	0.7199	4.1493	0.0001	0.0004	1.4068	up
LINC01504	-0.6782	-4.1315	0.0001	0.0004	1.3414	down
FGF22	-0.7595	-4.1313	0.0001	0.0004	1.3404	down
TMC5	0.5666	4.1295	0.0001	0.0004	1.3340	up
MMP19	0.6162	4.1289	0.0001	0.0004	1.3317	up
IL10RA	0.5599	4.1190	0.0001	0.0004	1.2954	up
IL22	0.5183	4.1168	0.0001	0.0004	1.2873	up
CD274	0.5149	4.1144	0.0001	0.0004	1.2785	up
BUB1B	0.6853	4.1097	0.0001	0.0004	1.2612	up
CPXM1	0.7831	4.1086	0.0001	0.0004	1.2572	up
BCHE	-0.5932	-4.0992	0.0001	0.0005	1.2228	down
FMNL2	0.6302	4.0988	0.0001	0.0005	1.2213	up
VWA3A	0.5587	4.0902	0.0001	0.0005	1.1900	up
FAM20A	0.5114	4.0902	0.0001	0.0005	1.1898	up
MPEG1	0.6079	4.0884	0.0001	0.0005	1.1834	up
DHRS9	0.7757	4.0832	0.0001	0.0005	1.1645	up
NAP1L3	-0.5762	-4.0812	0.0001	0.0005	1.1571	down
C1QTNF7	-0.6108	-4.0809	0.0001	0.0005	1.1559	down
DES	-1.3615	-4.0792	0.0001	0.0005	1.1496	down
PRRG3	-0.7351	-4.0674	0.0001	0.0005	1.1067	down

SIGLEC9	0.5602	4.0662	0.0001	0.0005	1.1025	up
PCP4L1	0.8185	4.0564	0.0001	0.0005	1.0670	up
TRBV20-1	0.6693	4.0556	0.0001	0.0005	1.0640	up
FAXDC2	-0.5668	-4.0501	0.0001	0.0005	1.0439	down
OSGIN1	0.5374	4.0394	0.0001	0.0006	1.0053	up
VMO1	0.5179	4.0310	0.0001	0.0006	0.9751	up
C9orf152	-0.5062	-4.0249	0.0001	0.0006	0.9531	down
GPR183	0.5954	4.0184	0.0001	0.0006	0.9296	up
PDE4B	0.5104	4.0041	0.0001	0.0006	0.8783	up
RTN4RL1	-0.5069	-3.9962	0.0001	0.0006	0.8497	down
SLC46A2	-0.8283	-3.9951	0.0001	0.0006	0.8459	down
GGT5	0.6050	3.9745	0.0001	0.0007	0.7723	up
CARD10	0.5031	3.9654	0.0001	0.0007	0.7398	up
EXO1	0.5100	3.9636	0.0001	0.0007	0.7333	up
PLEK	0.7073	3.9534	0.0001	0.0007	0.6972	up
LCE1B	-0.6434	-3.9422	0.0001	0.0008	0.6574	down
LILRB2	0.6134	3.9341	0.0001	0.0008	0.6289	up
CD200R1	0.7171	3.9315	0.0001	0.0008	0.6197	up
XAF1	0.6144	3.9297	0.0001	0.0008	0.6132	up
P2RY6	0.5826	3.9141	0.0001	0.0008	0.5581	up
AOAH	0.6376	3.9126	0.0001	0.0008	0.5530	up
HMGCS2	-0.8968	-3.9077	0.0001	0.0008	0.5355	down
GK	0.5390	3.9021	0.0001	0.0009	0.5160	up
CCKBR	-0.5058	-3.8966	0.0001	0.0009	0.4966	down
ICAM1	0.5958	3.8875	0.0001	0.0009	0.4647	up
TBX15	-0.6034	-3.8826	0.0002	0.0009	0.4475	down
PHLDA2	0.5909	3.8784	0.0002	0.0009	0.4329	up
SELP	0.5225	3.8744	0.0002	0.0009	0.4187	up
CLCA4	-0.6386	-3.8679	0.0002	0.0010	0.3963	down
FGFBP1	0.6345	3.8633	0.0002	0.0010	0.3801	up
CD3D	0.6195	3.8566	0.0002	0.0010	0.3567	up
COL15A1	0.5871	3.8534	0.0002	0.0010	0.3459	up
SH2D1A	0.6308	3.8504	0.0002	0.0010	0.3353	up
CRYM	-0.5625	-3.8362	0.0002	0.0011	0.2860	down
PSG4	-1.2173	-3.8348	0.0002	0.0011	0.2811	down
APMAP	-0.5447	-3.8197	0.0002	0.0011	0.2289	down
C1orf162	0.5530	3.8053	0.0002	0.0012	0.1793	up
TRDN	-0.5001	-3.7992	0.0002	0.0012	0.1582	down
ODF3L1	-0.7057	-3.7987	0.0002	0.0012	0.1567	down
IGSF3	0.5457	3.7966	0.0002	0.0012	0.1493	up
MS4A7	0.5625	3.7899	0.0002	0.0012	0.1263	up
TIMP3	-0.5649	-3.7893	0.0002	0.0012	0.1243	down
TSPAN8	-0.7747	-3.7809	0.0002	0.0012	0.0956	down
CARD18	-0.6665	-3.7768	0.0002	0.0013	0.0815	down

GJB4	-0.7447	-3.7728	0.0002	0.0013	0.0679	down
LIPG	0.6134	3.7688	0.0002	0.0013	0.0540	up
MS4A6A	0.6512	3.7642	0.0002	0.0013	0.0384	up
SPINK7	0.9638	3.7619	0.0002	0.0013	0.0306	up
CD93	0.5733	3.7576	0.0002	0.0013	0.0161	up
IKZF3	0.5760	3.7501	0.0002	0.0014	-0.0096	up
ADAMDEC1	0.7100	3.7426	0.0003	0.0014	-0.0352	up
POSTN	-0.8261	-3.7376	0.0003	0.0014	-0.0520	down
DOK2	0.5445	3.7336	0.0003	0.0014	-0.0658	up
APOBEC3G	0.5170	3.7309	0.0003	0.0015	-0.0748	up
PLAUR	0.6143	3.7286	0.0003	0.0015	-0.0824	up
C1QC	0.6760	3.7257	0.0003	0.0015	-0.0924	up
ATP6AP1L	-0.6170	-3.7136	0.0003	0.0015	-0.1332	down
ABCB1	0.5014	3.7123	0.0003	0.0015	-0.1375	up
PALM	-0.5215	-3.7061	0.0003	0.0016	-0.1585	down
SLC7A4	-0.5598	-3.7018	0.0003	0.0016	-0.1729	down
PTPN7	0.5451	3.6928	0.0003	0.0016	-0.2033	up
CCL17	0.8369	3.6900	0.0003	0.0016	-0.2128	up
IGFBP5	-0.5029	-3.6868	0.0003	0.0017	-0.2234	down
MSR1	0.7104	3.6842	0.0003	0.0017	-0.2321	up
LCE1C	-0.5760	-3.6742	0.0003	0.0017	-0.2657	down
HS3ST3B1	0.6015	3.6691	0.0003	0.0018	-0.2825	up
KCNK15	0.5048	3.6687	0.0003	0.0018	-0.2838	up
KRT39	-0.5740	-3.6673	0.0003	0.0018	-0.2886	down
PDCD1LG2	0.5172	3.6574	0.0003	0.0018	-0.3217	up
C1QB	0.7251	3.6562	0.0004	0.0018	-0.3255	up
CD2	0.7359	3.6378	0.0004	0.0019	-0.3867	up
FCGR2A	0.7100	3.6302	0.0004	0.0020	-0.4118	up
PI16	-0.9227	-3.6228	0.0004	0.0020	-0.4364	down
SLC27A2	-0.7556	-3.6226	0.0004	0.0020	-0.4372	down
SPRR3	0.5483	3.6207	0.0004	0.0020	-0.4434	up
DPP6	-0.5049	-3.5959	0.0004	0.0022	-0.5251	down
TRBC1	0.5880	3.5915	0.0004	0.0022	-0.5395	up
SLITRK6	-0.6085	-3.5911	0.0004	0.0022	-0.5407	down
LYPD2	0.6953	3.5882	0.0004	0.0022	-0.5503	up
PLA2R1	-0.5068	-3.5831	0.0005	0.0023	-0.5669	down
WNT16	-0.5080	-3.5678	0.0005	0.0024	-0.6168	down
IL1F10	-0.5769	-3.5652	0.0005	0.0024	-0.6256	down
ENHO	-0.5088	-3.5587	0.0005	0.0024	-0.6466	down
CD163	0.6850	3.5565	0.0005	0.0025	-0.6537	up
FCER1G	0.5632	3.5401	0.0005	0.0026	-0.7071	up
IGFL3	-0.5387	-3.5388	0.0005	0.0026	-0.7111	down
CYP3A4	-0.5589	-3.5381	0.0005	0.0026	-0.7134	down
ACOT12	-0.5268	-3.5192	0.0006	0.0027	-0.7745	down

MATN4	-0.7928	-3.5165	0.0006	0.0028	-0.7834	down
PAMR1	-0.5778	-3.4993	0.0006	0.0029	-0.8387	down
BCL2A1	0.5343	3.4883	0.0006	0.0030	-0.8738	up
IGFL4	0.5810	3.4836	0.0006	0.0031	-0.8887	up
SLC5A5	0.5088	3.4824	0.0006	0.0031	-0.8926	up
TIMP1	0.5142	3.4705	0.0007	0.0032	-0.9307	up
CD177	0.7629	3.4694	0.0007	0.0032	-0.9340	up
CACNA2D1	-0.5516	-3.4646	0.0007	0.0032	-0.9494	down
LINC01615	0.6290	3.4588	0.0007	0.0033	-0.9678	up
PCP4	-0.8216	-3.4525	0.0007	0.0033	-0.9877	down
SLC36A1	0.6940	3.4368	0.0008	0.0035	-1.0374	up
LAMP3	0.5094	3.4310	0.0008	0.0036	-1.0558	up
LRG1	0.8859	3.4166	0.0008	0.0037	-1.1013	up
SNX20	0.5774	3.4133	0.0008	0.0037	-1.1115	up
C1R	0.5061	3.4056	0.0008	0.0038	-1.1357	up
ALOX5AP	0.5105	3.4044	0.0008	0.0038	-1.1396	up
KRT2	-0.7325	-3.3968	0.0009	0.0039	-1.1633	down
BRI3	-0.5023	-3.3900	0.0009	0.0040	-1.1845	down
PTPRC	0.5223	3.3846	0.0009	0.0041	-1.2013	up
ANLN	0.6439	3.3842	0.0009	0.0041	-1.2026	up
NDC1	0.6572	3.3483	0.0010	0.0045	-1.3141	up
GSTM5	-0.6653	-3.3470	0.0010	0.0045	-1.3180	down
SCARA5	-0.7393	-3.3239	0.0011	0.0048	-1.3892	down
CCL19	0.7337	3.3167	0.0011	0.0049	-1.4113	up
SPINT3	-0.7144	-3.3149	0.0011	0.0050	-1.4167	down
FGFBP2	-0.5407	-3.2885	0.0012	0.0053	-1.4973	down
PPP1R3C	0.5163	3.2736	0.0013	0.0055	-1.5427	up
BATF	0.5394	3.2683	0.0013	0.0056	-1.5587	up
DKK2	-0.5215	-3.2542	0.0014	0.0059	-1.6013	down
DCN	-0.5482	-3.2479	0.0014	0.0060	-1.6204	down
FPR1	0.5928	3.2421	0.0015	0.0061	-1.6377	up
SGCA	-0.6353	-3.2419	0.0015	0.0061	-1.6382	down
CRISP3	0.5915	3.2066	0.0016	0.0067	-1.7440	up
ELF5	0.9663	3.1969	0.0017	0.0068	-1.7726	up
DCT	-0.5361	-3.1897	0.0017	0.0070	-1.7940	down
MYH11	-0.6253	-3.1707	0.0018	0.0073	-1.8501	down
CCL2	0.6104	3.1525	0.0019	0.0077	-1.9036	up
KCNQ5	0.6348	3.1476	0.0020	0.0078	-1.9180	up
NAP1L2	-0.5234	-3.1313	0.0021	0.0081	-1.9655	down
PGLYRP3	0.5077	3.0950	0.0023	0.0090	-2.0709	up
CXCL8	0.8582	3.0852	0.0024	0.0092	-2.0991	up
SPINK9	0.5625	3.0722	0.0025	0.0095	-2.1364	up
KRT9	0.9461	3.0613	0.0026	0.0098	-2.1677	up
AQP5	0.9182	3.0450	0.0027	0.0102	-2.2141	up

TF	-0.6186	-3.0141	0.0030	0.0111	-2.3016	down
SLC28A3	0.5484	2.9684	0.0035	0.0125	-2.4293	up
MMP1	0.8916	2.9656	0.0035	0.0125	-2.4371	up
DDX60L	0.5387	2.9578	0.0036	0.0128	-2.4588	up
ETV4	0.6208	2.9539	0.0036	0.0129	-2.4697	up
S100A3	0.5904	2.9298	0.0039	0.0137	-2.5362	up
CNN1	-0.8083	-2.9102	0.0041	0.0144	-2.5897	down
ACTA1	-0.6694	-2.8888	0.0044	0.0153	-2.6480	down
BIRC3	0.5551	2.8816	0.0045	0.0155	-2.6676	up
CD300E	0.9177	2.8681	0.0047	0.0161	-2.7041	up
APOC1	-0.6706	-2.8545	0.0049	0.0166	-2.7405	down
H2AC20	0.5926	2.8536	0.0049	0.0166	-2.7430	up
INHBA	0.5551	2.8042	0.0057	0.0188	-2.8746	up
MYO3A	-0.6325	-2.7872	0.0060	0.0196	-2.9192	down
SEC14L4	-0.5426	-2.7588	0.0065	0.0210	-2.9936	down
MS4A4A	0.5074	2.7490	0.0067	0.0215	-3.0191	up
DNER	0.8015	2.7247	0.0072	0.0228	-3.0820	up
CYSLTR1	-0.5028	-2.7046	0.0076	0.0239	-3.1335	down
CXCL10	0.7321	2.6594	0.0087	0.0266	-3.2482	up
DGAT2L6	-1.1841	-2.6435	0.0091	0.0276	-3.2881	down
SPINK6	0.6061	2.6432	0.0091	0.0276	-3.2889	up
COCH	-0.6595	-2.6152	0.0098	0.0295	-3.3585	down
SUSD4	0.5206	2.6149	0.0098	0.0295	-3.3593	up
IGHA1	-0.7189	-2.6097	0.0100	0.0298	-3.3722	down
WFDC3	-0.5984	-2.6007	0.0102	0.0305	-3.3944	down
ADH1B	-0.5478	-2.5931	0.0104	0.0310	-3.4131	down
ACTG2	-0.7677	-2.5874	0.0106	0.0314	-3.4271	down
PPP1R1B	-0.5341	-2.5656	0.0112	0.0330	-3.4804	down
CYP1A1	-0.5471	-2.5483	0.0118	0.0342	-3.5225	down
IFIH1	0.5118	2.5164	0.0129	0.0368	-3.5993	up
KCNK5	0.5448	2.4998	0.0135	0.0381	-3.6389	up
FADS2	-1.1491	-2.4718	0.0145	0.0406	-3.7051	down
ELOVL3	-1.1134	-2.4680	0.0147	0.0409	-3.7140	down
PDZK1	-0.5052	-2.4173	0.0168	0.0457	-3.8321	down
AADACL3	-0.9588	-2.3873	0.0182	0.0486	-3.9009	down
LCE1E	-0.5358	-2.3747	0.0188	0.0499	-3.9296	down

Table S3. GSEA of atopic dermatitis.

Description	setSize	Enrichment Score	NES	Padj	FDR
CHEMOKINE_SIGNALING_PATHWAY	176	0.533	2.013	0.044	0.033
CELL_CYCLE	123	0.551	1.966	0.044	0.033
CYTOKINE_CYTOKINE_RECEPTOR_INTERACTION	227	0.504	1.955	0.044	0.033

FRUCTOSE_AND_MANNOSE_METABOLISM	34	0.669	1.937	0.044	0.033
PYRIMIDINE_METABOLISM	94	0.556	1.893	0.047	0.035
PROTEASOME	42	0.611	1.874	0.044	0.033
RIG_I_LIKE_RECEPTOR_SIGNALING_PATHWAY	54	0.586	1.869	0.044	0.033
INTESTINAL_IMMUNE_NETWORK_FOR_IGA_PRODUCTION	40	0.597	1.807	0.044	0.033
LEISHMANIA_INFECTION	67	0.551	1.785	0.047	0.035
GLYCOSPHINGOLIPID_BIOSYNTHESIS_LACTO_AND_NEOLACTO_SERIES	25	0.655	1.778	0.044	0.033
AMINO_SUGAR_AND_NUCLEOTIDE_SUGAR_METABOLISM	43	0.568	1.754	0.047	0.035
NATURAL_KILLER_CELL_MEDIATED_CYTOTOXICITY	109	0.482	1.684	0.047	0.035
TIGHT_JUNCTION	117	-0.420	-1.622	0.045	0.033
RIBOSOME	86	-0.582	-2.132	0.045	0.033

Table S4. GO enrichment pathway.

ONTOLOGY	ID	Description	p.adjust	qvalue	geneID	Count
BP	GO:0042451	purine nucleoside biosynthetic process	1.21E-02	0.0017	AMD1	1
BP	GO:0042455	ribonucleoside biosynthetic process	1.21E-02	0.0017	AMD1	1
BP	GO:0046129	purine ribonucleoside biosynthetic process	1.21E-02	0.0017	AMD1	1
BP	GO:0046512	sphingosine biosynthetic process	1.21E-02	0.0017	SPTLC2	1
BP	GO:0046520	sphingoid biosynthetic process	1.21E-02	0.0017	SPTLC2	1
BP	GO:0006596	polyamine biosynthetic process	1.21E-02	0.0017	AMD1	1
BP	GO:0006684	sphingomyelin metabolic process	1.21E-02	0.0017	SPTLC2	1
BP	GO:0009163	nucleoside biosynthetic process	1.21E-02	0.0017	AMD1	1
BP	GO:0034404	nucleobase-containing small molecule biosynthetic process	1.21E-02	0.0017	AMD1	1
BP	GO:0006595	polyamine metabolic process	1.21E-02	0.0017	AMD1	1
BP	GO:0006670	sphingosine metabolic process	1.21E-02	0.0017	SPTLC2	1
BP	GO:1901659	glycosyl compound biosynthetic process	1.21E-02	0.0017	AMD1	1
BP	GO:0034312	diol biosynthetic process	1.21E-02	0.0017	SPTLC2	1
BP	GO:0046128	purine ribonucleoside metabolic process	1.21E-02	0.0017	AMD1	1
BP	GO:0046519	sphingoid metabolic process	1.21E-02	0.0017	SPTLC2	1

BP	GO:0042278	purine nucleoside metabolic process	1.40E-02	0.0019	AMD1	1
BP	GO:0034311	diol metabolic process	1.47E-02	0.0020	SPTLC2	1
BP	GO:0009119	ribonucleoside metabolic process	1.64E-02	0.0022	AMD1	1
BP	GO:0042401	cellular biogenic amine biosynthetic process	1.64E-02	0.0022	AMD1	1
BP	GO:0009309	amine biosynthetic process	1.64E-02	0.0022	AMD1	1
BP	GO:0060612	adipose tissue development	1.69E-02	0.0023	SPTLC2	1
BP	GO:0035272	exocrine system development	1.77E-02	0.0024	IGSF3	1
BP	GO:0009116	nucleoside metabolic process	2.16E-02	0.0029	AMD1	1
BP	GO:0046173	polyol biosynthetic process	2.16E-02	0.0029	SPTLC2	1
BP	GO:0016239	positive regulation of macroautophagy	2.16E-02	0.0029	SPTLC2	1
BP	GO:0046513	ceramide biosynthetic process	2.16E-02	0.0029	SPTLC2	1
BP	GO:0061912	selective autophagy	2.17E-02	0.0030	SPTLC2	1
BP	GO:1901657	glycosyl compound metabolic process	2.71E-02	0.0037	AMD1	1
BP	GO:0006576	cellular biogenic amine metabolic process	2.86E-02	0.0039	AMD1	1
BP	GO:0006672	ceramide metabolic process	2.86E-02	0.0039	SPTLC2	1
BP	GO:0030148	sphingolipid biosynthetic process	2.86E-02	0.0039	SPTLC2	1
BP	GO:0044106	cellular amine metabolic process	2.97E-02	0.0041	AMD1	1
BP	GO:0019751	polyol metabolic process	2.97E-02	0.0041	SPTLC2	1
BP	GO:0009308	amine metabolic process	2.98E-02	0.0041	AMD1	1
BP	GO:0010508	positive regulation of autophagy	3.05E-02	0.0042	SPTLC2	1
BP	GO:0046165	alcohol biosynthetic process	3.21E-02	0.0044	SPTLC2	1
BP	GO:0016241	regulation of macroautophagy	3.21E-02	0.0044	SPTLC2	1
BP	GO:0046467	membrane lipid biosynthetic process	3.21E-02	0.0044	SPTLC2	1
BP	GO:0044272	sulfur compound biosynthetic process	3.26E-02	0.0044	AMD1	1
BP	GO:0006665	sphingolipid metabolic process	3.33E-02	0.0045	SPTLC2	1
BP	GO:0072522	purine-containing compound biosynthetic process	4.14E-02	0.0056	AMD1	1
BP	GO:0006643	membrane lipid metabolic process	4.14E-02	0.0056	SPTLC2	1
BP	GO:1901617	organic hydroxy compound biosynthetic process	4.71E-02	0.0064	SPTLC2	1
BP	GO:0061448	connective tissue development	4.80E-02	0.0065	SPTLC2	1
BP	GO:0008654	phospholipid biosynthetic process	4.80E-02	0.0065	SPTLC2	1

CC	GO:0002178	palmitoyltransferase complex	3.37E-03	NA	SPTLC2	1
CC	GO:0140534	endoplasmic reticulum protein-containing complex	1.91E-02	NA	SPTLC2	1
MF	GO:0016408	C-acyltransferase activity	1.52E-02	NA	SPTLC2	1
MF	GO:0016831	carboxy-lyase activity	1.52E-02	NA	AMD1	1
MF	GO:0016409	palmitoyltransferase activity	1.52E-02	NA	SPTLC2	1
MF	GO:0016830	carbon-carbon lyase activity	1.52E-02	NA	AMD1	1
MF	GO:0030170	pyridoxal phosphate binding	1.52E-02	NA	SPTLC2	1
MF	GO:0070279	vitamin B6 binding	1.52E-02	NA	SPTLC2	1
MF	GO:0019842	vitamin binding	3.43E-02	NA	SPTLC2	1
MF	GO:0016829	lyase activity	3.96E-02	NA	AMD1	1
MF	GO:0016747	acyltransferase activity, transferring groups other than amino-acyl groups	3.98E-02	NA	SPTLC2	1
MF	GO:0016746	acyltransferase activity	4.03E-02	NA	SPTLC2	1

Table S5. GeneMania GO enrichment pathway.

ONTOLOGY	ID	Description	p.adjust	qvalue	geneID	Count
BP	GO:0046500	S-adenosylmethionine metabolic process	3.45E-07	＜0.0001	MAT1A/MAT2A/MAT2B/MRI1	4
BP	GO:0006555	methionine metabolic process	3.45E-07	＜0.0001	SMS/MAT1A/MTAP/MRI1	4
BP	GO:0044272	sulfur compound biosynthetic process	1.32E-06	＜0.0001	AMD1/MAT1A/MAT2A/MAT2B/MTAP/MRI1	6
BP	GO:0000096	sulfur amino acid metabolic process	4.34E-06	＜0.0001	SMS/MAT1A/MTAP/MRI1	4
BP	GO:0006790	sulfur compound metabolic process	4.34E-06	＜0.0001	AMD1/SMS/MAT1A/MAT2A/MAT2B/MTAP/MRI1	7
BP	GO:0009066	aspartate family amino acid metabolic process	1.31E-05	＜0.0001	SMS/MAT1A/MTAP/MRI1	4
BP	GO:0006596	polyamine biosynthetic process	1.95E-05	＜0.0001	AMD1/SMS/SRM	3
BP	GO:0046513	ceramide biosynthetic process	3.09E-05	＜0.0001	SPTLC2/SPTLC1/SPTSSA	4

					/SPTSSB	
BP	GO:0006595	polyamine metabolic process	3.38E-05	＜0.0001	AMD1/SMS/SRM	3
BP	GO:1901605	alpha-amino acid metabolic process	7.11E-05	＜0.0001	SMS/MAT1A/MTAP/MRI1/CTPS1	5
BP	GO:0006672	ceramide metabolic process	1.31E-04	0.0001	SPTLC2/SPTLC1/SPTSSA/SPTSSB	4
BP	GO:0030148	sphingolipid biosynthetic process	1.31E-04	0.0001	SPTLC2/SPTLC1/SPTSSA/SPTSSB	4
BP	GO:0042401	cellular biogenic amine biosynthetic process	2.20E-04	0.0001	AMD1/SMS/SRM	3
BP	GO:0009309	amine biosynthetic process	2.21E-04	0.0001	AMD1/SMS/SRM	3
BP	GO:0006730	one-carbon metabolic process	2.42E-04	0.0001	MAT1A/MAT2A/MAT2B	3
BP	GO:0006520	cellular amino acid metabolic process	2.77E-04	0.0002	SMS/MAT1A/MTAP/MRI1/CTPS1	5
BP	GO:0046467	membrane lipid biosynthetic process	3.29E-04	0.0002	SPTLC2/SPTLC1/SPTSSA/SPTSSB	4
BP	GO:0006665	sphingolipid metabolic process	4.38E-04	0.0002	SPTLC2/SPTLC1/SPTSSA/SPTSSB	4
BP	GO:0042451	purine nucleoside biosynthetic process	9.19E-04	0.0005	AMD1/MTAP	2
BP	GO:0042455	ribonucleoside biosynthetic process	9.19E-04	0.0005	AMD1/MTAP	2
BP	GO:0046129	purine ribonucleoside biosynthetic process	9.19E-04	0.0005	AMD1/MTAP	2
BP	GO:0006643	membrane lipid metabolic process	1.01E-03	0.0006	SPTLC2/SPTLC1/SPTSSA/SPTSSB	4
BP	GO:0009086	methionine biosynthetic process	1.01E-03	0.0006	MTAP/MRI1	2
BP	GO:0046512	sphingosine biosynthetic process	1.09E-03	0.0006	SPTLC2/SPTLC1	2
BP	GO:0046520	sphingoid biosynthetic process	1.09E-03	0.0006	SPTLC2/SPTLC1	2
BP	GO:0009163	nucleoside biosynthetic process	1.36E-03	0.0007	AMD1/MTAP	2

BP	GO:0034404	nucleobase-containing small molecule biosynthetic process	1.36E-03	0.0007	AMD1/MTAP	2
BP	GO:0000097	sulfur amino acid biosynthetic process	1.70E-03	0.0009	MTAP/MRI1	2
BP	GO:0006576	cellular biogenic amine metabolic process	1.92E-03	0.0011	AMD1/SMS/SRM	3
BP	GO:0006670	sphingosine metabolic process	1.92E-03	0.0011	SPTLC2/SPTLC1	2
BP	GO:1901659	glycosyl compound biosynthetic process	1.92E-03	0.0011	AMD1/MTAP	2
BP	GO:0009067	aspartate family amino acid biosynthetic process	2.09E-03	0.0011	MTAP/MRI1	2
BP	GO:0034312	diol biosynthetic process	2.09E-03	0.0011	SPTLC2/SPTLC1	2
BP	GO:0046128	purine ribonucleoside metabolic process	2.09E-03	0.0011	AMD1/MTAP	2
BP	GO:0046519	sphingoid metabolic process	2.09E-03	0.0011	SPTLC2/SPTLC1	2
BP	GO:0044106	cellular amine metabolic process	2.26E-03	0.0012	AMD1/SMS/SRM	3
BP	GO:0009308	amine metabolic process	2.49E-03	0.0014	AMD1/SMS/SRM	3
BP	GO:0051291	protein heterooligomerization	2.74E-03	0.0015	MAT2A/ROM1	2
BP	GO:0042278	purine nucleoside metabolic process	2.82E-03	0.0016	AMD1/MTAP	2
BP	GO:0043094	cellular metabolic compound salvage	2.82E-03	0.0016	MTAP/MRI1	2
BP	GO:0034311	diol metabolic process	3.43E-03	0.0019	SPTLC2/SPTLC1	2
BP	GO:0046825	regulation of protein export from nucleus	3.59E-03	0.0020	XPO1/TXN	2
BP	GO:0009119	ribonucleoside metabolic process	4.77E-03	0.0026	AMD1/MTAP	2
BP	GO:0006611	protein export from nucleus	1.23E-02	0.0068	XPO1/TXN	2
BP	GO:0009116	nucleoside metabolic process	1.39E-02	0.0077	AMD1/MTAP	2
BP	GO:0046173	polyol biosynthetic process	1.39E-02	0.0077	SPTLC2/SPTLC1	2
BP	GO:0016239	positive regulation of macroautophagy	1.41E-02	0.0077	SPTLC2/SPTLC1	2
BP	GO:0051259	protein complex oligomerization	1.46E-02	0.0080	MAT1A/MAT2A/ROM1	3

BP	GO:0061912	selective autophagy	1.54E-02	0.0084	SPTLC2/SPTLC1	2
BP	GO:1901607	alpha-amino acid biosynthetic process	1.54E-02	0.0084	MTAP/MRI1	2
BP	GO:0008652	cellular amino acid biosynthetic process	1.87E-02	0.0103	MTAP/MRI1	2
BP	GO:1901657	glycosyl compound metabolic process	2.45E-02	0.0135	AMD1/MTAP	2
BP	GO:1990830	cellular response to leukemia inhibitory factor	2.73E-02	0.0150	SRM/MAT2A	2
BP	GO:1990823	response to leukemia inhibitory factor	2.73E-02	0.0150	SRM/MAT2A	2
BP	GO:0046822	regulation of nucleocytoplasmic transport	3.32E-02	0.0183	XPO1/TXN	2
BP	GO:0019751	polyol metabolic process	3.75E-02	0.0206	SPTLC2/SPTLC1	2
BP	GO:0010508	positive regulation of autophagy	4.33E-02	0.0238	SPTLC2/SPTLC1	2
CC	GO:0002178	palmitoyltransferase complex	1.03E-08	＜0.0001	SPTLC2/SPTLC1/SPTSSA/SPTSSB	4
CC	GO:0140534	endoplasmic reticulum protein-containing complex	1.39E-04	0.0001	SPTLC2/SPTLC1/SPTSSA/SPTSSB	4
MF	GO:0016408	C-acyltransferase activity	3.76E-07	＜0.0001	SPTLC2/SPTLC1/SPTSSA/SPTSSB	4
MF	GO:0016409	palmitoyltransferase activity	2.53E-06	＜0.0001	SPTLC2/SPTLC1/SPTSSA/SPTSSB	4
MF	GO:0016765	transferase activity, transferring alkyl or aryl (other than methyl) groups	1.07E-05	＜0.0001	SMS/MAT1A/SRM/MAT2A	4
MF	GO:0016747	acyltransferase activity, transferring groups other than amino-acyl groups	1.63E-03	0.0009	SPTLC2/SPTLC1/SPTSSA/SPTSSB	4
MF	GO:0016746	acyltransferase activity	2.06E-03	0.0012	SPTLC2/SPTLC1/SPTSSA/SPTSSB	4
MF	GO:0016879	ligase activity, forming carbon-nitrogen bonds	1.27E-02	0.0073	CTPS1/NADSYN1	2
MF	GO:0030170	pyridoxal phosphate binding	1.29E-02	0.0075	SPTLC2/SPTLC1	2

MF	GO:0070279	vitamin B6 binding	1.29E-02	0.0075	SPTLC2/SPTLC1	2

Table S6. GeneMania KEGG enrichment pathway.

Pathway	Count	P_{adj}-Value
Cysteine and methionine metabolism	8	0.0000
Biosynthesis of cofactors	5	0.0001
Sphingolipid signaling pathway	4	0.0006
Arginine and proline metabolism	3	0.0007
Biosynthesis of amino acids	3	0.0018
Sphingolipid metabolism	2	0.0169
Glutathione metabolism	2	0.0169
